# A biomedical perspective of pyocyanin from *Pseudomonas aeruginosa*: its applications and challenges

**DOI:** 10.1007/s11274-024-03889-0

**Published:** 2024-02-10

**Authors:** Samriti Balaji Mudaliar, Alevoor Srinivas Bharath Prasad

**Affiliations:** Department of Public Health & Genomics, Manipal School of Life Sciences (MSLS), Manipal Academy of Higher Education (MAHE), Manipal, Karnataka 576104 India

**Keywords:** Antibacterial, Anticancer, Antifungal, Neuroprotective, *Pseudomonas aeruginosa*, Pyocyanin

## Abstract

Pyocyanin is a bioactive pigment produced by *Pseudomonas aeruginosa*. It is an important virulence factor that plays a critical role in *P. aeruginosa* infections as a redox-active secondary metabolite and a quorum sensing (QS) signaling molecule. Pyocyanin production from chorismic acid requires the involvement of two homologous operons, *phz1* and *phz2*, which are activated by QS regulatory proteins. Pyocyanin inhibits the proliferation of bacterial, fungal, and mammalian cells by inducing oxidative stress due to which it acts as a potent antibacterial, antifungal, and anticancer agent. Its potential role as a neuroprotectant needs further exploration. However, pyocyanin exacerbates the damaging effects of nosocomial infections caused by *P. aeruginosa* in immunocompromised individuals. Further, cystic fibrosis (CF) patients are highly susceptible to persistent *P. aeruginosa* infections in the respiratory system. The bacterial cells form colonies and three interconnected QS networks—*pqs, las*, and *rhl*—get activated, thus stimulating the cells to produce pyocyanin which exacerbates pulmonary complications. As an opportunistic pathogen, *P. aeruginosa* produces pyocyanin to impede the recovery of injuries like burn wounds through its anti-proliferative activity. Moreover, pyocyanin plays a vital role in compounding *P. aeruginosa* infections by promoting biofilm formation. This review begins with a brief description of the characteristics of pyocyanin, its activity, and the different aspects of its production including its biosynthesis, the role of QS, and the effect of environmental factors. It then goes on to explore the potential applications of pyocyanin as a biotherapeutic molecule while also highlighting the biomedical challenges and limitations that it presents.

**Graphical abstract:**

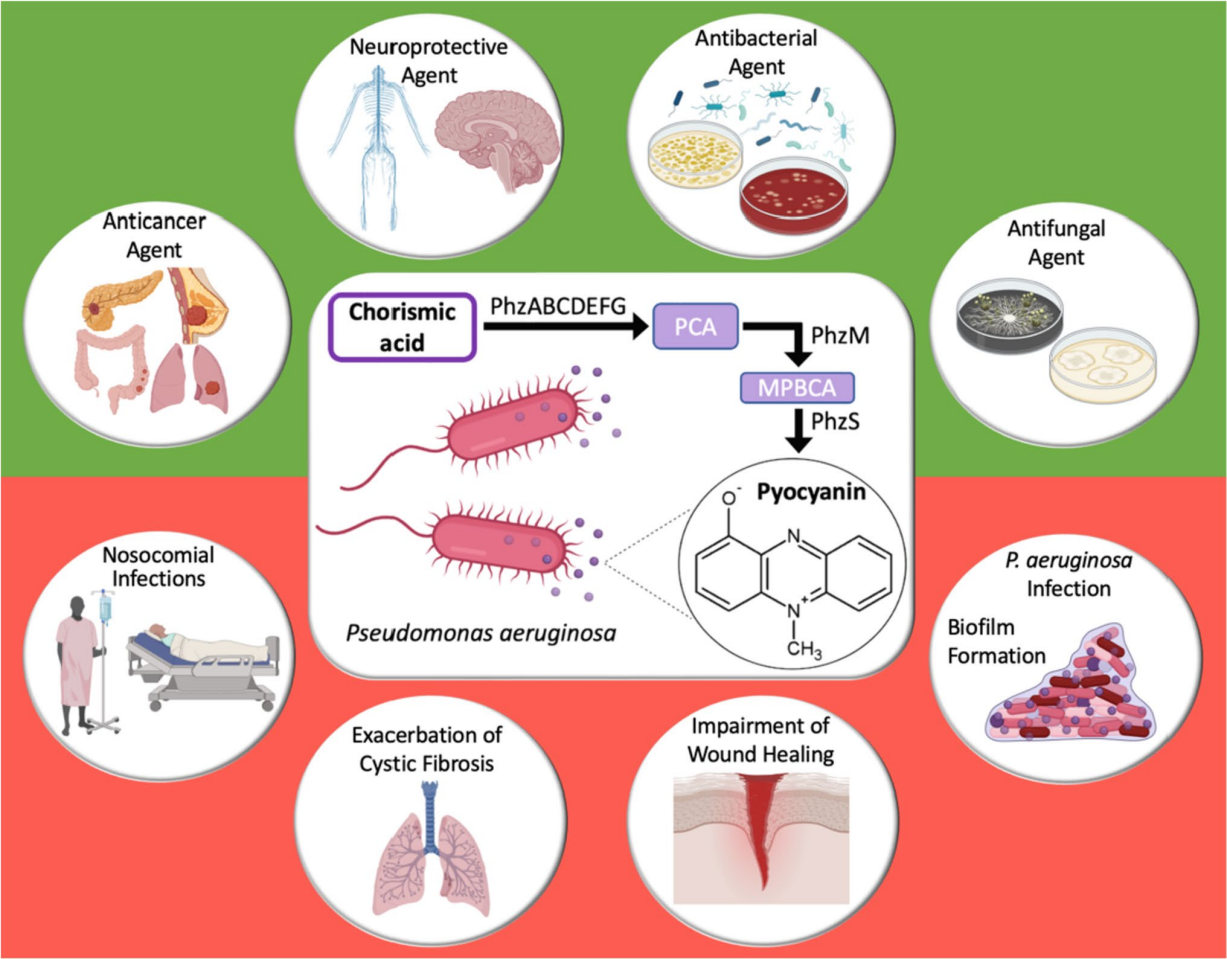

## Introduction

**Fig. 1:**
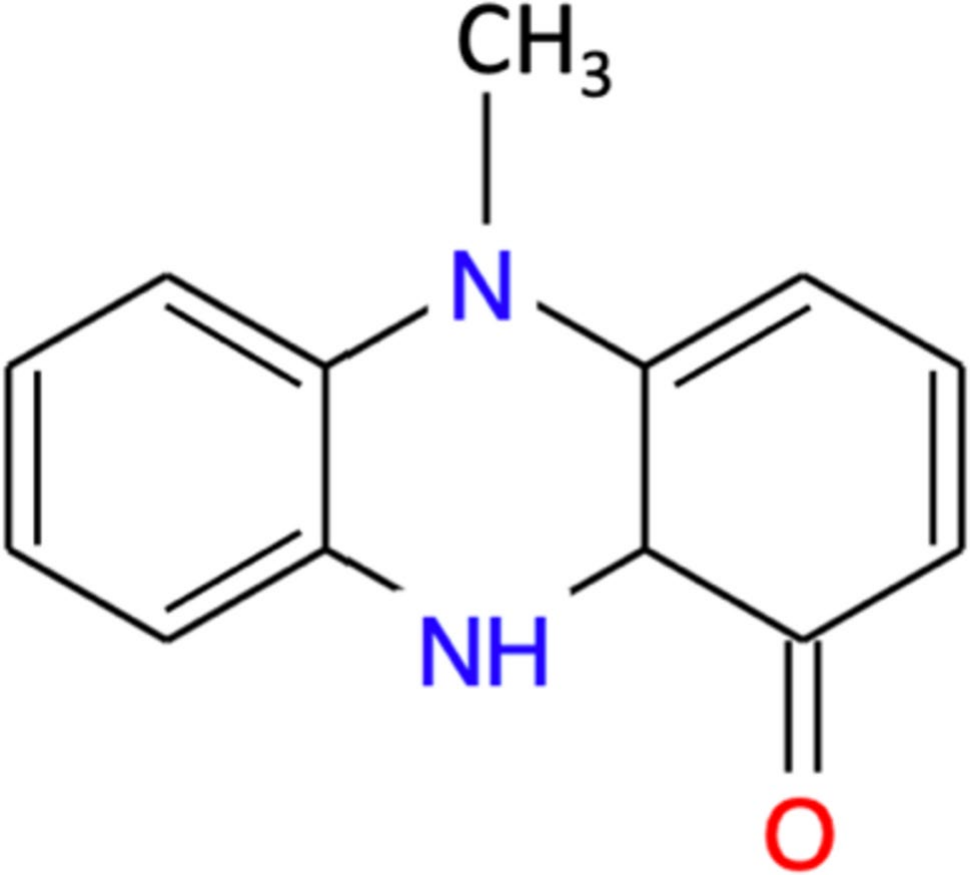
Chemical structure of pyocyanin

Pyocyanin is a water-soluble, blue-green, redox-active pigment produced exclusively by 95% of the strains of *Pseudomonas aeruginosa*, a Gram-negative, aerobic, rod-shaped bacterium. *P. aeruginosa* is commercially important in the generation of several biotechnological products such as lipases, proteases, and rhamnolipids. Pyocyanin is one such bioactive compound produced by *P. aeruginosa* that has a plethora of applications including its use as a natural chromogenic dye in the textile industry and a biocontrol agent in agriculture (Srivastava et al. 2022). Pyocyanin was first extracted in 1860 from blue-tinged surgical dressings infected with *P. aeruginosa* by Fordos (1863) who studied its physical properties including its solubility in different solvents and the pH-specific variations in its color. Since the pigment imparts a blue color to pus as well as wound dressings, it was named ‘pyocyanin’ based on Greek terms denoting the words ‘pus’ and ‘blue’. In 1882, while attempting to uncover the parasitic cause for the blue coloration of infected wounds, Gessard managed to directly isolate pyocyanin from *P. aeruginosa*, then known as ‘pyocyanic bacillus’ or ‘blue pus bacillus’. Upon its isolation in 1924, pyocyanin became the first natural phenazine to be obtained in the laboratory in its pure form (Turner and Messenger 1986; Gonçalves and Vasconcelos 2021). As correctly elucidated by Hillemann in 1938, pyocyanin is a heterocyclic compound with the chemical formula 1-hydroxy-5-methyl phenazine (C_13_H_10_N_2_O), shown in Fig. 1 (Schoental 1941). The crystal structure of pyocyanin has been elucidated and it is classified as a nitrogen-containing phenazine derivative. Each molecule of the bacterial pigment comprises two subunits of *N*-methyl-1-hydroxyphenazine (Jayaseelan et al. 2014). Credited with coining the term ‘antibiotic’ in 1942, Walksman (1973) was the first to put forward the therapeutic potential of pyocyanin as an antibiotic formulation known as ‘pyocyanase’. However, despite possessing several beneficial properties that can be translated into biomedical therapies, pyocyanin is yet to be clinically introduced for therapeutic use due to its role in the virulence and pathogenesis of *P. aeruginosa*.

**Fig. 2:**
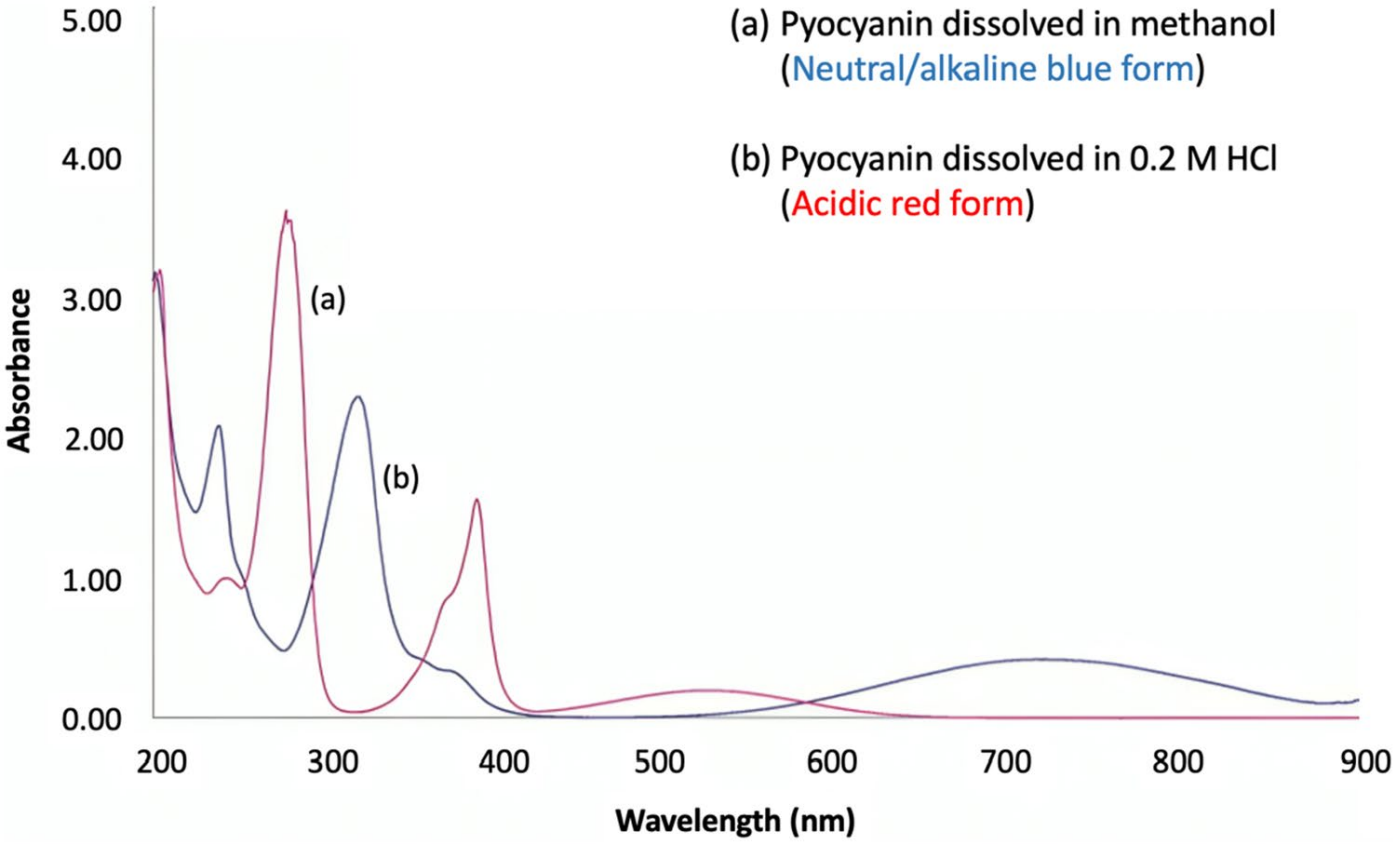
UV–Vis absorption spectrum of pyocyanin (Ohfuji et al. 2004)

Pyocyanin is a redox-active molecule that appears bright blue in color in neutral or alkaline conditions since it exists in the oxidized form but its color changes to a deep red in acidic conditions as it undergoes protonation (Sutradhar et al. 2018). The corresponding shift in the UV–Vis absorption spectra of pyocyanin due to its redox activity has been graphically represented in Fig. 2. At a neutral pH of 7.0, pyocyanin generally exists in the zwitterionic form due to which it appears blue in color (Ohfuji et al. 2004).

Pyocyanin is one of the most important secondary metabolites produced by *Pseudomonas aeruginosa* since it acts as a virulence factor and a quorum sensing (QS) signaling molecule (Jayaseelan et al. 2014). Further, it is an electron acceptor which is of importance to the bacterial cells in maintaining redox balance. Pyocyanin facilitates the survival of *P. aeruginosa* despite oxidant limitations by removing excess electrons during anaerobic as well as microaerophilic conditions (Rada and Leto 2013). Moreover, pyocyanin exhibits antibacterial activity by disrupting the cell membrane-associated respiratory chain and can inhibit biofilm formation by other microbes. Up to 95% of *P. aeruginosa*’s antimicrobial properties are attributed to pyocyanin (Jayaseelan et al. 2014). It is also a powerful antifungal agent since it can interfere with the electron transport chain of several fungi (Srivastava et al. 2022). Thus, pyocyanin is critical for the endurance and proliferation of *P. aeruginosa.*

The activity of pyocyanin is influenced by pyoverdine, another important secondary metabolite produced by *P. aeruginosa*. Pyoverdine is a siderophore with a high affinity for binding to ferric (Fe^3+^) ions due to which it plays a major role in scavenging iron for the survival and growth of the bacterial cells in iron-deficient conditions (Durán et al. 2022). Pyocyanin is involved in stable biofilm formation while pyoverdine-mediated iron uptake enhances bacterial growth within the biofilm thus leading to the increased production of pyocyanin. Further, the oxidative damage caused to the neighboring bacterial and fungal cells by pyocyanin increases the availability of ferric ions thus facilitating the acquisition of iron by pyoverdine. However, in its reduced form, pyocyanin reduces ferric (Fe^3+^) ions to ferrous (Fe^2+^) ions thus hindering iron uptake by pyoverdine (Koley et al. 2011). AlgR is a transcriptional regulator that co-ordinates the expression of pyocyanin and pyoverdine, thus affecting the virulence of *P. aeruginosa*. It has been experimentally shown that, in the constitutively unphosphorylated state, AlgR increases pyocyanin production as compared to the wild-type AlgR while it enhances pyoverdine production in its phosphorylated form. Additionally, its control over pyoverdine is carbon-dependent while pyocyanin production is unaffected by the media used (Little et al. 2018).

**Fig. 3:**
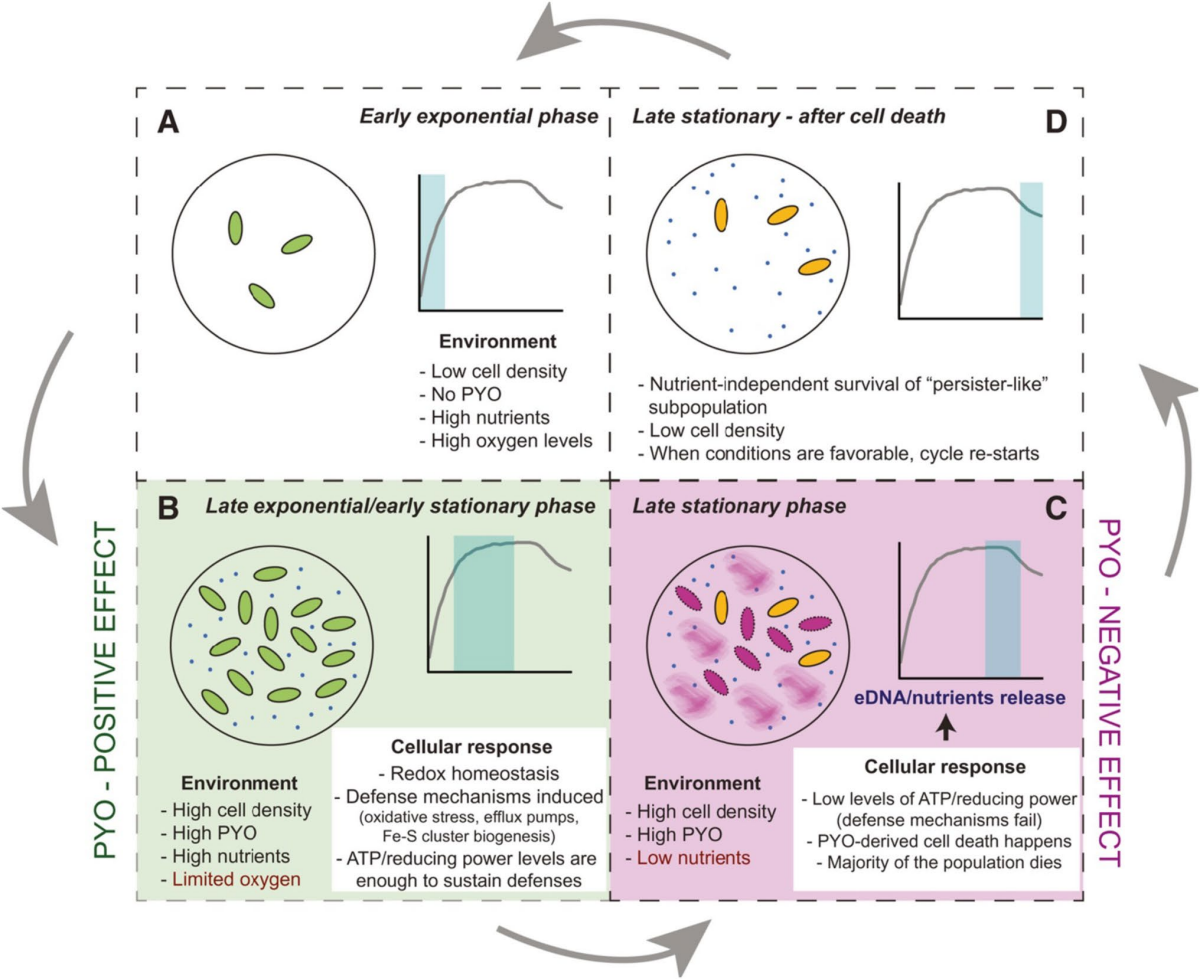
Positive and negative effects of pyocyanin throughout the *P. aeruginosa* life cycle (Meirelles and Newman 2018)

While pyocyanin provides several benefits to *P. aeruginosa*, it also has certain negative effects due to which it is known as a ‘double-edged sword’ (Meirelles and Newman 2018). In order to avoid auto-poisoning by pyocyanin, bacterial cells employ several ATP-dependent defense strategies. One strategy is the oxidative stress response and antioxidant defense by upregulating the activity of detoxifying enzymes like superoxide dismutase (SOD) and catalase. Another defense mechanism is the biogenesis of Fe–S clusters, important prosthetic groups necessary for proper protein functioning, in order to replace the Fe–S groups damaged due to increased ROS production. However, in conditions of limited ATP production either due to the inhibition of ATP synthase or due to nutrient limitation and carbon shortage, these defense mechanisms fail and pyocyanin becomes auto-toxic to a majority of the *P. aeruginosa* cells, especially at high concentrations, causing pyocyanin-mediated cell death followed by the release of extracellular DNA (eDNA). Only a small fraction of the cells, known as ‘persister-like’ cells, can intrinsically resist the virulent action of pyocyanin at high concentrations even in nutrition-deficient environments (Meirelles and Newman 2018). Fig. 3 summarizes the beneficial and detrimental effects of pyocyanin on *P. aeruginosa* throughout its life cycle.

Given the diverse range of properties possessed by pyocyanin as well as its ambiguous nature, it is a molecule of great interest among researchers. It is used as a natural reagent for dyeing textiles and fabrics and as a biocontrol agent in agricultural and animal husbandry (DeBritto et al. 2020). It also finds application in microbial fuel cells (MFCs) for the generation of green energy by enhancing toluene biodegradation (Wu et al. 2014). Several works of literature have explored the various aspects involved in the biosynthesis of pyocyanin and the optimal parameters required to maximize its production, isolation, and purification (El-Fouly et al. 2015; Abdelaziz et al. 2023). The history, virulence mechanisms, and potential biotechnological applications of pyocyanin in different industries have been elucidated by Gonçalves and Vasconcelos (2021) and Sahoo et al. (2023). A number of scientific articles have reviewed the physical, chemical, and biological properties of pyocyanin as well as its beneficial characteristics and undesirable properties (Jayaseelan et al. 2014; Rani and Azmi 2019; Srivastava et al. 2022; Jabłońska et al. 2023). This review, on the other hand, offers a biomedical perspective on pyocyanin which assumes great importance given the fact that pyocyanin is yet to be used as a biotherapeutic despite its unending potential due to its virulent nature and its role in the exacerbation of *P. aeruginosa* infections in immunocompromised individuals, skin abrasions and wounds, as well as cystic fibrosis patients. The review draws focus to the medically useful properties of pyocyanin while also highlighting the adverse effects of pyocyanin and the risks of using it in healthcare. It presents both the beneficial and detrimental effects of pyocyanin in the field of biomedicine in order to provide a nuanced understanding of the molecule. Through this review, we hope to impress upon the need for future research into the formulation and implementation of pyocyanin-targeted therapies as well as the utilization of pyocyanin itself as a potent biotherapeutic molecule. The review begins with a brief description of the various facets involved in the production of pyocyanin, including the organisms that produce pyocyanin, its biosynthetic pathway, the role played by QS in its synthesis, and finally, the influence of various environmental factors on its production. Subsequently, it delves into the potential biomedical applications of pyocyanin given its ability to induce oxidative stress in several species, with a special emphasis on its anticancer, neuroprotective, antibacterial, and antifungal properties which have been illustrated in detail. This is followed by an explanation of the biomedical challenges posed by pyocyanin including its detrimental role in nosocomial infections, cystic fibrosis, wound healing, and *P. aeruginosa* infections. Finally, the review discusses the limitations that hinder the introduction of pyocyanin as a biomedical therapeutic molecule.

## Production of pyocyanin

*Pseudomonas aeruginosa* is the only microorganism that naturally produces pyocyanin which imparts a bluish-green color to the bacterial cultures. Around 95% of *P. aeruginosa* strains, such as PAO1 and PA14, are able to produce pyocyanin due to the expression of *phzM* and *phzS,* the two novel, phenazine-specific genes necessary for the conversion of PCA to pyocyanin (Muller and Merrett 2014; Saleem et al. 2021). Though *phzS* encodes for the phzS enzyme in other *Pseudomonas* species like *P. fluorescens* as well as other bacteria like *Escherichia coli*, these organisms cannot synthesize pyocyanin in the absence of phzM and instead convert PCA directly to 1-hydroxyphenazine. Further, it was experimentally shown that the insertional inactivation of either *phzM* or *phzS* in *P. aeruginosa* inhibited pyocyanin production. In the case of *Pseudomonas chlororaphis*, a different phenazine-modifying gene known as *phzH* is expressed which converts PCA into phenazine-1-carboxamide instead of pyocyanin (Mavrodi et al. 2001). Therefore, not all *Pseudomonas* species can produce pyocyanin despite the activation of the *phz1* and *phz2* operons. However, microbes that are not natural pyocyanin producers can be metabolically engineered to synthesize high concentrations of pyocyanin. The first successful attempt at genetically manipulating a heterologous microbe to produce pyocyanin was accomplished by co-transforming chemically competent *E. coli* cells with two plasmid constructs, one containing the genes necessary for the conversion of chorismic acid to PCA and the other comprising the genes needed to convert PCA into pyocyanin (da Silva et al. 2021).

### Biosynthetic pathway for pyocyanin production

**Fig. 4:**
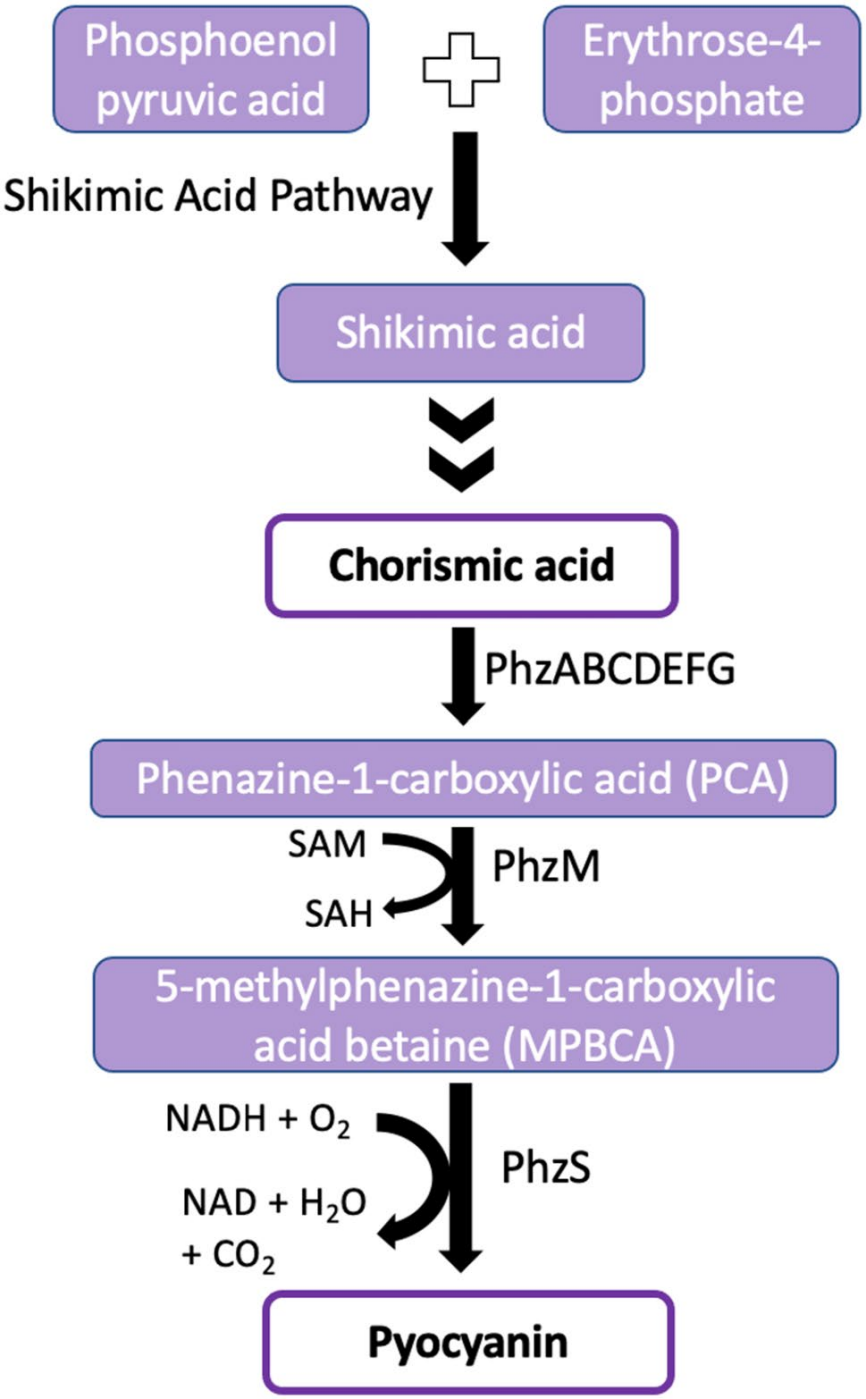
Biosynthetic pathway for the production of pyocyanin by *P. aeruginosa*

The biosynthesis of pyocyanin, as illustrated in Fig. 4, begins with the precursor, chorismic acid (CA), which is derived from shikimic acid, the end-product of the shikimic acid pathway (Jayaseelan et al. 2014). The conversion of chorismic acid to phenazine-1-carboxylic acid (PCA) involves seven, phenazine-specific, highly conserved, enzyme-encoding genes, namely, *phzA*, *phzB*, *phzC*, *phzD*, *phzE*, *phzF*, and *phzG*, that are comprised within two homologous core biosynthetic loci viz., *phzA*_*1*_*B*_*1*_*C*_*1*_*D*_*1*_*E*_*1*_*F*_*1*_*G*_*1*_ (*phz1*) and *phzA*_*2*_*B*_*2*_*C*_*2*_*D*_*2*_*E*_*2*_*F*_*2*_*G*_*2*_ (*phz2*) operons (Sterritt et al. 2018). The subsequent conversion of PCA to 5-methyl phenazine-1-carboxylic acid betaine (MPCBA) requires an additional gene, *phzM*, which encodes the enzyme phzM, an S-adenosylmethionine (SAM) dependent phenazine-specific methyltransferase that catalyzes the attachment of a methyl group to the nitrogen-phenazine group of PCA. Finally, pyocyanin is obtained by the decarboxylation of MPCBA through the involvement of the *phzS* gene which codes for phzS, a FAD-dependent monooxygenase enzyme (Parsons et al. 2007). Thus, *phzM* and *phzS* are the two critical phenazine-modifying genes necessary for pyocyanin biosynthesis and their inactivation leads to pyocyanin deficiency. In cases of PCA accumulation due to a decrease in the expression of *phzM*, the excess PCA is directly converted to 1-hydroxyphenazine by the action of the phzS enzyme. In fact, contrary to the previous hypothesis that pyocyanin is decomposed by light to form 1-hydroxyphenazine, it has been experimentally proved using *Escherichia coli* that exogenous PCA is directly converted to 1-hydroxyphenazine by the enzymatic activity of phzS (Mavrodi et al. 2001).

### Role of quorum sensing (QS) in pyocyanin production

**Fig. 5:**
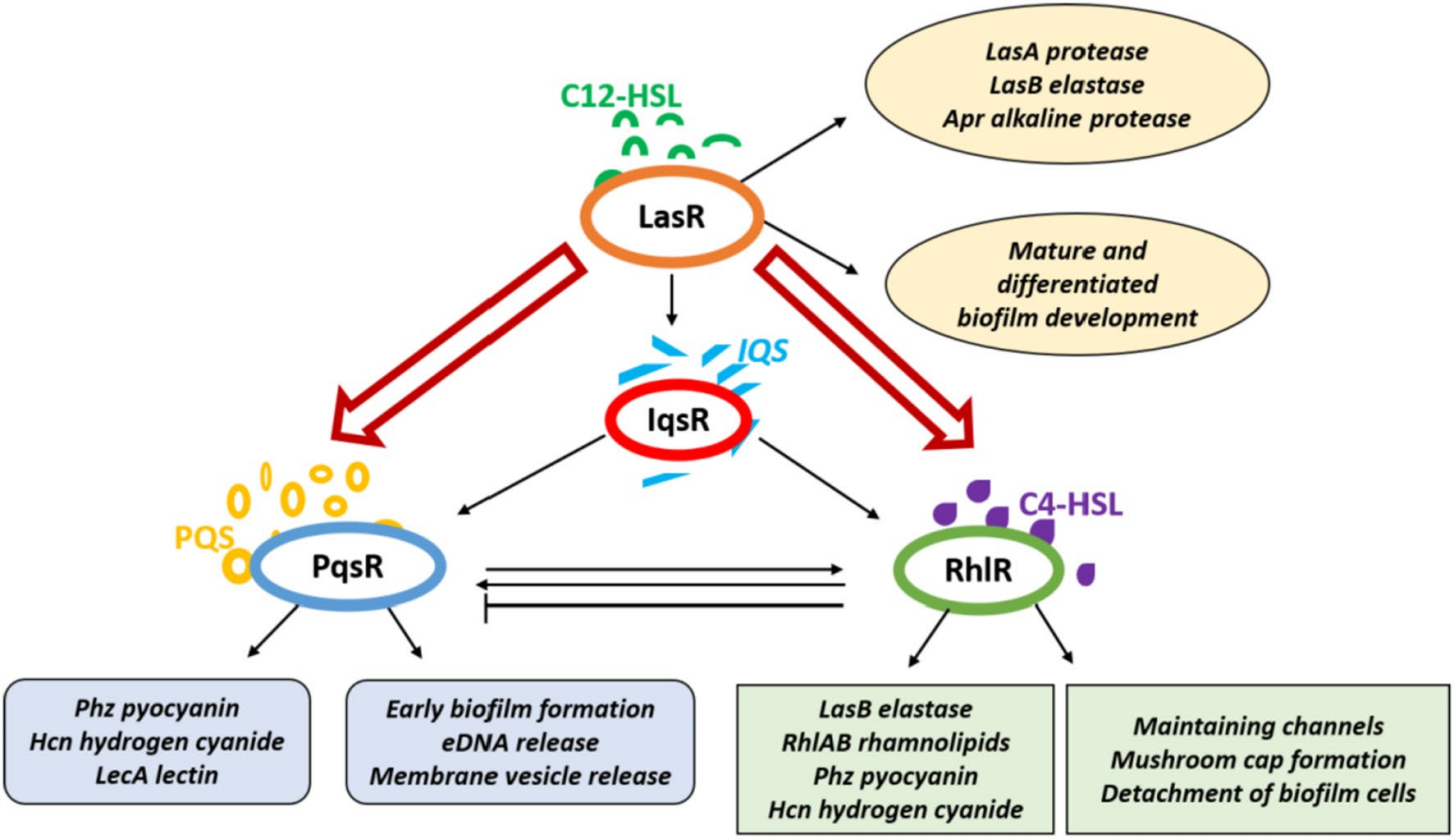
Quorum sensing (QS) mechanism of *P. aeruginosa* (Vetrivel et al. 2021)

The production of pyocyanin is controlled by the quorum sensing (QS) mechanism of *P. aeruginosa* which comprises four main interconnected systems viz., *las*, *pqs*, *rhl*, and the recently discovered *integrated QS *(*IQS*), as displayed in Fig. 5. These systems produce signaling molecules that bind to their specific signal receptors to regulate the expression of genes involved in biofilm formation and virulence. In the *las* system, LasI synthase synthesizes the signaling molecule, *N*-(3-oxododecanoyl)-l-homoserine lactones (C12-HSL), which binds to the LasR receptor. Similarly, the signaling molecule 2-heptyl-3-hydroxy-4-quinolone (PQS) recognizes PqsR in *pqs*, RhlI synthase produces *N*-butanoyl-l-homoserine lactone (C4-HSL) which binds to RhlR in *rhl*, and finally, the *IQS* system releases the autoinducer 2-(2-hydroxyphenyl)-thiazole-4-carbaldehyde which is specific to IqsR. The *las* system activates *IQS* by regulating its autoinducer. Subsequently, *las* and *IQS* regulate *pqs* as well as *rhl*, which is itself activated by the PQS molecule. Furthermore, RhlR expression is controlled by LasR which is the first receptor to be activated (Vetrivel et al. 2021). The regulatory proteins, *RhlR* and *PqsE*, from the *rhl* and *pqs* networks respectively, jointly activate the two operons, *phz1* and *phz2*, necessary for pyocyanin biosynthesis (Higgins et al. 2018). Pyocyanin functions as one of the final signaling molecules in the QS cascade of *P. aeruginosa* and affects its colony characteristics (Dietrich et al. 2006). It has been experimentally demonstrated that pyocyanin-producing wild-type colonies display a smooth morphology for a longer duration as compared to the non-pyocyanin-producing mutant colonies which show a wrinkled appearance much faster. Moreover, colonies that overproduce pyocyanin were shown to maintain a smooth surface for the entire duration of the study (Dietrich et al. 2006).

### Effect of environmental factors on pyocyanin production

The rate of production of pyocyanin varies depending on several environmental factors including the bacterial source, nitrogen source, carbon source, temperature, pH, as well as the presence of different compounds. The results of an experimental study comparing the concentration of pyocyanin produced by *P. aeruginosa* strains isolated from different environmental sources and clinical samples revealed that maximum production was observed in strains isolated from rice-cultivated soil and urinary tract infection samples (El-Fouly et al. 2015). It has also been shown that peptone is the optimal nitrogen source for maximizing pyocyanin production. Other organic nitrogen sources like beef extract and urea as well as inorganic sources including ammonium sulfate [(NH_4_)_2_SO_4_] and ammonium chloride (NH_4_Cl) also facilitate a comparable level of pyocyanin production (El-Fouly et al. 2015; Gahlout et al. 2021). The presence of a carbon source significantly increases the pigment concentration with maximum synthesis seen in the case of sugar alcohols like glycerol and mannitol, potentially because glycerol and alanine together form a precursor for pyocyanin production (Frank and De Moss 1958). The use of maltose as a carbon source also facilitates the release of a notable concentration of pyocyanin (Gahlout et al. 2021). In the case of urine isolates of *P. aeruginosa*, an increased rate of pigment production was observed at an incubation temperature of 37 °C with continuous shaking at around 200 rpm. Pyocyanin production was seen to begin at 10 hours post-incubation and reach a maximum level at 72 hours (Elbargisy 2021). The most favorable pH for pyocyanin production is slightly alkaline ranging from 7.4 to 8.4 while extreme conditions having a pH value either above 9.0 or below 6.0 are highly unsuitable (Abdelaziz et al. 2023). Pyocyanin biosynthesis is also affected by the compounds present in the environment surrounding the bacterial colonies. Studies have demonstrated that inorganic salts like sodium chloride (NaCl) and calcium chloride (CaCl_2_) as well as sources of iron including ferric chloride (FeCl_3_) and ferric sulphate (FeSO_4_) greatly improve pigment levels (Mathew et al. 2011; Özcan and Kahraman 2015; Gahlout et al. 2021). The presence of organic solvents like toluene and chloroform as well as surfactants such as Triton X-100 and Tween 20 has also been experimentally proven to enhance pyocyanin synthesis (Ozdal 2019). However, certain compounds including nitric oxide, nanomaterials like silver nanoparticles (Ag NPs) and zinc oxide nanoparticles (ZnO NPs), phenolic compounds such as quercetin and ellagic acid, sodium citrate, benzimidazolium salts, and antimicrobial peptides like calgranulin C have been shown to inhibit the synthesis of pyocyanin, possible by interfering with the expression of *phzM* (Zhou et al. 2022; Jabłońska et al. 2023).

## Biomedical applications of pyocyanin

**Fig. 6:**
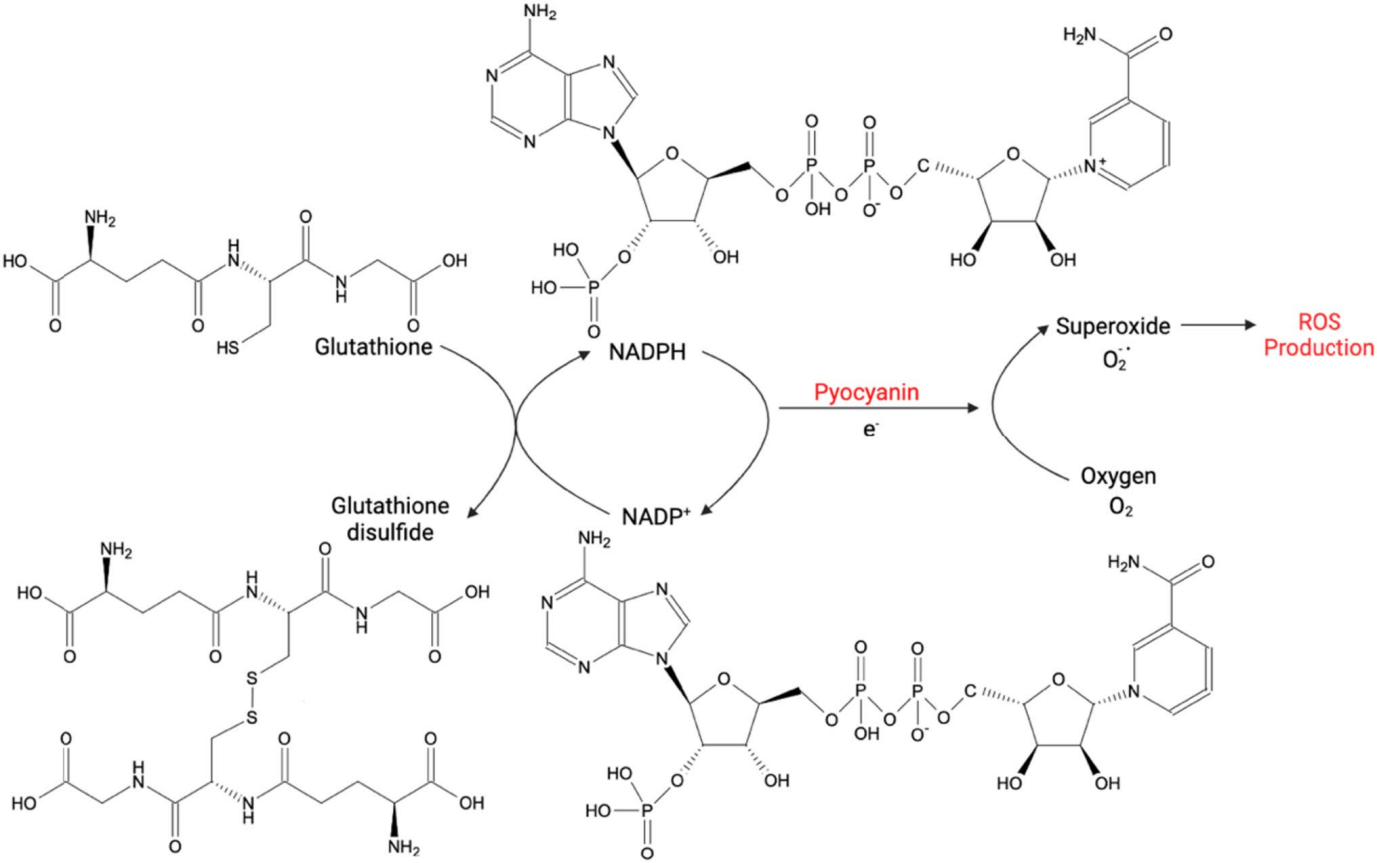
Production of ROS by pyocyanin

Pyocyanin is known for its ability to inhibit the cellular growth of different bacterial, fungal, and mammalian species. The proposed mechanism of action is that having a low molecular weight of 210 Da zwitterion, pyocyanin can easily diffuse through the cell membrane, and as a redox-active biomolecule, it can generate oxidative stress by increasing the intracellular levels of reactive oxygen species (ROS) like superoxide (O_2_^**.**−^) and hydrogen peroxide (H_2_O_2_). ROS generation is brought about by the simultaneous oxidation of glutathione (GSH) to glutathione disulfide (GSSG) and the reduction of NADP^+^ to NADPH followed by the enzymatic reduction of pyocyanin. Fig. 6 demonstrates the process of intracellular ROS production by pyocyanin.

The production of ROS by pyocyanin leads to oxidative stress in bacterial, fungal, and mammalian cells which causes several negative effects including a decrease in NADPH levels, inhibition of essential enzymes, DNA damage, disruption of membrane potential, oxidative damage of the components involved in cell cycle and its regulation (Abdelaziz et al. 2023). Thus, the cytotoxic effects due to increased ROS production by pyocyanin ultimately lead to cell death followed by cell lysis (O'Malley et al. 2003a). This ability of pyocyanin greatly contributes to its potential as a therapeutic agent in medicine and healthcare. Further, pyocyanin has also been shown to hinder the activities of cellular acetylcholinesterase (AChE) and 5-lipoxygenase which are overexpressed in neurodegenerative disorders, thus indicating its possible neuroprotective effect. Table 1 encapsulates the potential biomedical applications of pyocyanin.

**Table 1:** Potential biomedical applications of pyocyanin

Application	Activity of pyocyanin	References
Anticancer agent	• Demonstrates highly specific cytotoxicity towards cancer cells • IC_50_ value varies with the type of cancer • Exhibits concentration-dependent anti-proliferative effect • Depletes cellular ATP, inhibits aconitase activity, and activates caspase-3 which cleaves cell cycle regulatory proteins • Creates oxidative stress via ROS generation leading to DNA damage in cancer cells • Destroys cancer cells through apoptosis and necrosis	O’Malley et al. (2003a), Zhao et al. (2014), Patil et al. (2017), Moayedi et al. (2018), Koyun et al. (2022), Abdelaziz et al. (2022)
Neuroprotective agent	• Inhibits the enzymatic activity of cellular acetylcholinesterase (AChE) which is overexpressed in neurodegenerative disorders • Shows significant anti-AChE activity as compared to currently used AChE inhibitors such as genistein and huperzine-A • Reduces the activity of 5-lipoxygenase down to 30% thereby hindering the production of compounds like leukotriene B_4_ which are elevated in neurodegenerative disorders	Muller and Sorrell (1991), Chu and Praticò (2016), Koyun et al. (2022), Abdelaziz et al. (2023)
Antibacterial agent	• Exhibits antimicrobial efficacy comparable to that of tetracycline against a wide range of pathogenic Gram-positive and Gram-negative bacteria that are responsible for several diseases like urinary tract infections and gastrointestinal problems • Minimum inhibitory concentration (MIC) depends on the bacterial species • Impedes bacterial pathogenesis by hindering biofilm formation	Laxmi et al. (2016), Devnath et al. (2017); Hamad et al. (2020), Saleem et al. (2021), Kamer et al. (2023)
Antifungal agent	• Possesses concentration-dependent antifungal activity against several pathogenic fungi • MIC depends on the fungal species; MIC against *Trichophyton rubrum* is lower than that of the commercial standard, fluconazole • Inhibits fungal growth and proliferation by creating oxidative stress • Inhibits spore formation and causes hyphal swelling in *Fusarium oxysporum* which causes fusariosis	Hedayati et al. (2007), Alzahrani and Alqahtani (2016), Mahmoud et al. (2016), El-Zawawy and Ali (2016), Abdul-Hussien and Atia (2018)

### Pyocyanin as an anticancer agent

**Fig. 7:**
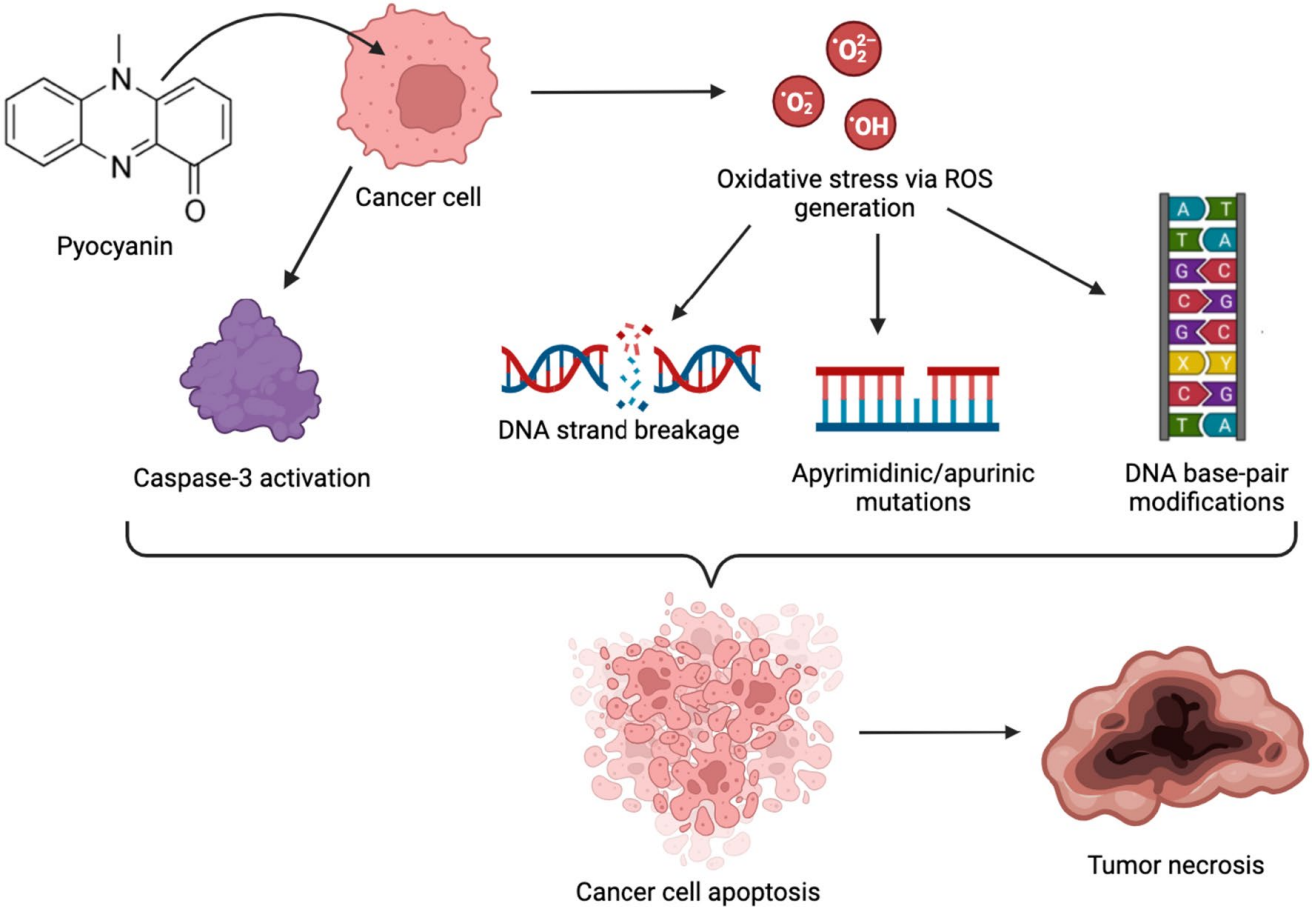
Anticancer property of pyocyanin

Cancer is one of the leading causes of mortality on a global scale with 10 million deaths recorded in 2020 alone and an expected 47% rise in cancer burden across the world within the next two decades (Sung et al. 2021). Pyocyanin is a potential chemotherapeutic agent as it has been reported to exhibit cytotoxic effects against different human cancer cell lines at a specific range of concentrations in several in vitro studies. Pyocyanin has the ability to inhibit the proliferation of cancer cells and decrease cell viability, with its anti-proliferative and cytotoxic properties increasing with an increase in concentration, making it a viable chemotherapeutic agent for combating several types of cancers. Zhao et al. (2014) experimentally showed the ability of pyocyanin to induce cytotoxicity in HepG2 human hepatoma cells via accelerated DNA damage and cell death. This is brought about by caspase 3 activation as well as oxidative stress due to increased ROS production by glutathione oxidation. Activated caspase 3 is necessary for apoptosis while oxidative stress causes apyrimidinic or apurinic mutations, base-pair modifications, and strand breaks, ultimately leading to DNA damage. Pyocyanin exhibited a similar concentration-dependent cytotoxic effect on the human pancreatic cancer cell line, PANC-1, by inducing apoptosis and necrosis. The observed anti-proliferative effect was again associated with ROS generation leading to oxidative stress. The mechanism of pyocyanin’s anticancer activity is outlined in Fig. 7. The IC_50_ value of pyocyanin displays a significant variation based on the clinical *Pseudomonas aeruginosa* strain used indicating that its activity differs based on the source of extraction (Moayedi et al. 2018).

**Fig. 8:**
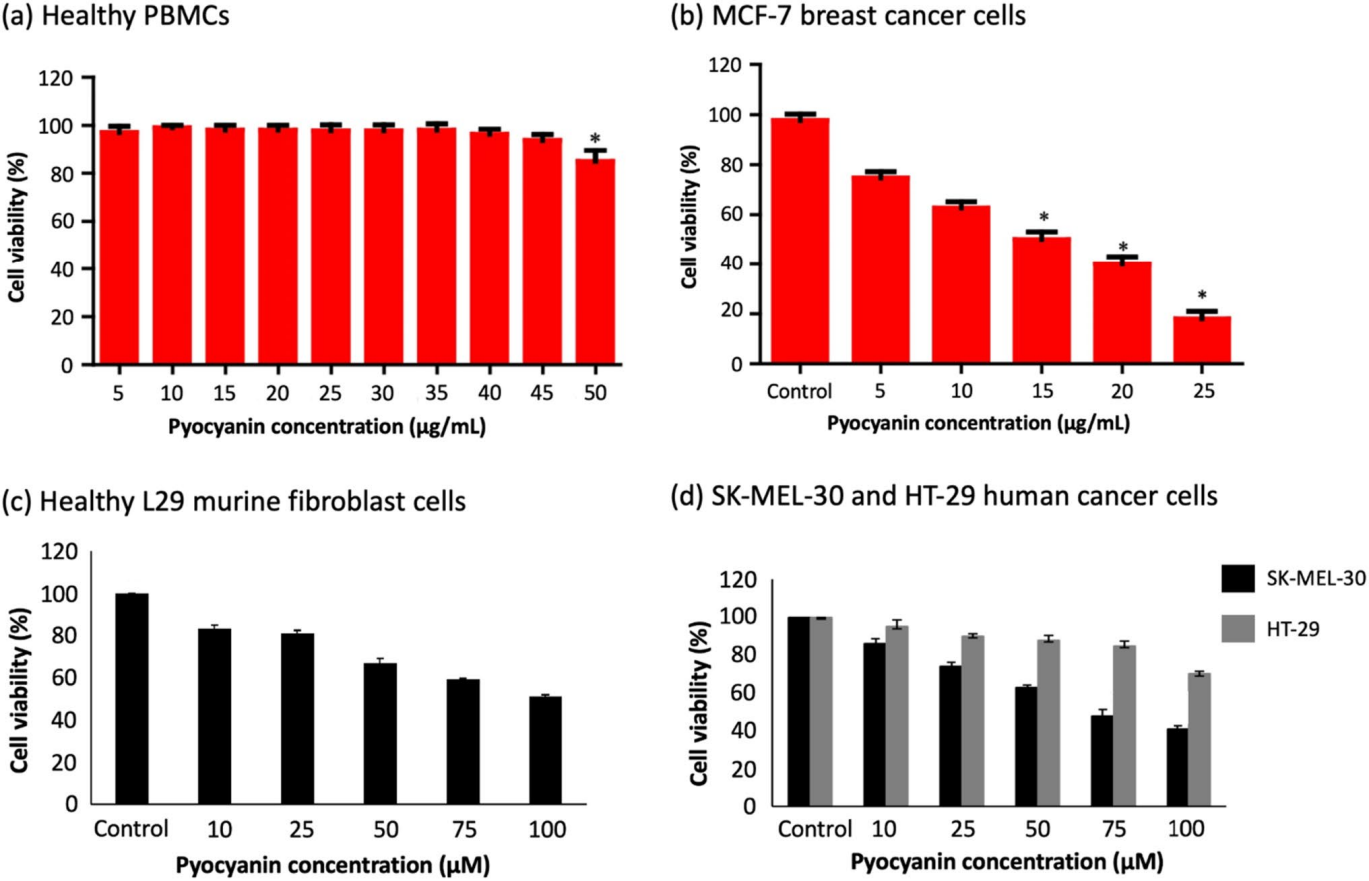
Graphs showing change in cell viability of different cell lines on increasing pyocyanin concentration (Abdelaziz et al. 2022; Koyun et al. 2022)

The anticancer effect of pyocyanin also varies based on the type of cancer as shown by Patil et al. (2017) in an experimental study wherein the concentration of pyocyanin required to completely inhibit cell growth was 160.29 μM in the case of A549 human lung carcinoma cells while a concentration of 269.22 μM was required for HeLa human cervical cancer cells. For KB human squamous cell carcinoma cells, only concentrations higher than 380.52 μM were able to inhibit cell proliferation. This variation in concentration has been attributed to the differences in metabolism, genetic composition, and physiology across cell lines. The cytotoxic effect of pyocyanin on A549 cells was previously investigated by O’Malley et al. (2003a) and it was confirmed that pyocyanin depletes cellular ATP and impedes aconitase activity possibly via depolarization of the mitochondrial membrane and subsequent overexpression of mitochondrial superoxide dismutase. The results of a study conducted by Narayani et al. (2021) demonstrated the cytotoxic potential of the bacterial pigment against the H-29 human colorectal adenocarcinoma cell line with an IC_50_ value of 215.42 μM. Pyocyanin also possesses the ability to control the spread of leukemia as demonstrated by its cytotoxic effect against HL-60, a human promyelocytic leukemia cell line, with an extremely low IC_50_ value of 5.82 μM (Kohatsu et al. 2020). Cytotoxicity analysis of pyocyanin in normal human cells is critical before progressing to the in vivo stage of experimentation since the pigment should not affect healthy cells in order to be used as a viable chemotherapeutic agent. Pyocyanin was also shown to be highly selective for lung cancer since its IC_50_ value in A549 cells was significantly lower than that in MRC-5 normal human lung fibroblast cells (Kohatsu et al. 2020). The results of a recent study conducted by Abdelaziz et al. (2022) showed the highly specific nature of pyocyanin’s anticancer activity against the MCF-7 human breast adenocarcinoma cell line with an IC_50_ value of 71.35 μM since the cell viability of normal human peripheral blood mononuclear cells (PBMCs) was not affected even on increasing the concentration of pyocyanin up to 237.82 μM. Pyocyanin was shown to induce apoptosis and necrosis in cancer cells via the activation of caspase-3 to cleave proteins involved in cell cycle regulation (Abdelaziz et al. 2022). Another study published by Koyun et al. (2022) established the concentration-dependent anti-proliferative activity of pyocyanin against HT-29 human colon cancer cells, decreasing the cell viability to 50% at 179 μM while its activity was significantly stronger against SK-MEL-30 human melanoma cells with a much lower IC_50_ value of 72 μM. However, the cell viability of healthy L929 murine fibroblast cells dropped to 51% on treatment with 100 μM of pyocyanin (Koyun et al. 2022). Figure 8 compares the change in cell viability of different healthy and cancerous cell lines upon increasing the concentration of pyocyanin. Therefore, further research into the selectivity of pyocyanin towards cancer cells must be confirmed through in vivo studies and clinical trials in order to utilize the cytotoxic capacity of pyocyanin in chemotherapeutic strategies.

### Pyocyanin as a neuroprotective agent

**Fig. 9:**
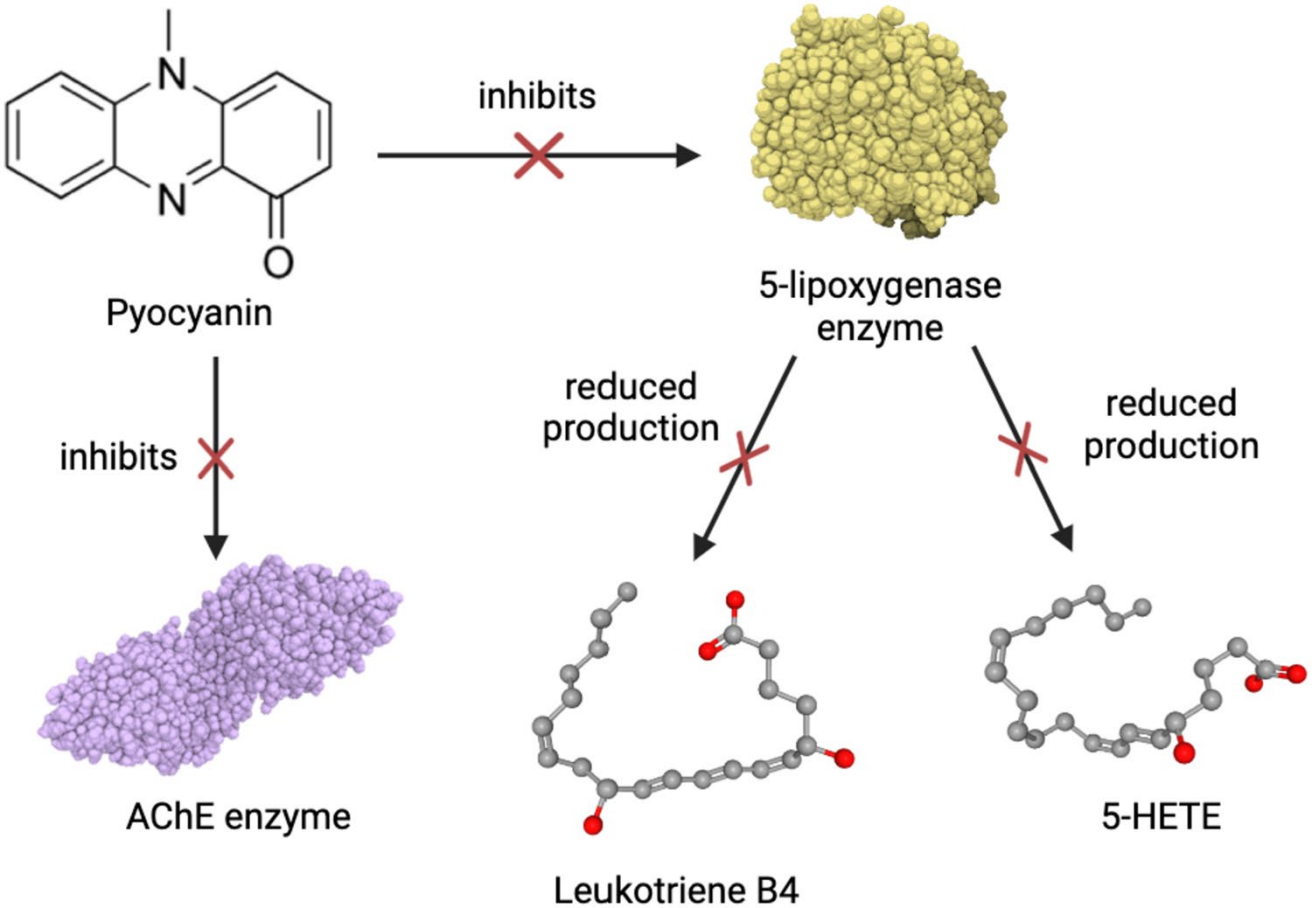
Neuroprotective property of pyocyanin

Neurodegenerative disorders comprise a major factor responsible for the high mortality rate among elderly human populations. They also significantly hinder the quality of life by disrupting cognitive functions, emotional balance, motor skills, and sleep patterns. Some of the most prevalent and detrimental neurodegenerative diseases include Alzheimer’s disease, amyotrophic lateral sclerosis (ALS), and Parkinson’s disease (Erkkinen et al. 2018). Pyocyanin is a potential neuroprotective agent due to its inhibitory effect on the enzymatic activity of cellular acetylcholinesterase (AChE). It is a promising treatment for neurodegenerative disorders characterized by elevated levels of the AChE enzyme which hydrolyses the neurotransmitter, acetylcholine. This impedes cholinergic conduction, ultimately resulting in the loss of neural function (Abdelaziz et al. 2023). The results of the novel study published by Koyun et al. (2022) showed that 100 μM of pyocyanin exhibits the highest inhibitory action against AChE activity in SH-SY5Y human neuroblastoma cells. The anti-AChE property of pyocyanin is attributed to its small size and zwitterionic nature which allows it to pass through cellular membranes in eukaryotes, as well as its unique redox chemistry. Reportedly, the activity of pyocyanin is comparable to that of currently used AChE inhibitors such as genistein and huperzine-A (Koyun et al. 2022). Thus, pyocyanin is a viable alternative to other AChE inhibitory compounds for the treatment of Alzheimer’s disease, myasthenia gravis, and other neurodegenerative disorders associated with reduced levels of acetylcholine. Moreover, pyocyanin has been shown to inhibit the activity of 5-lipoxygenase up to 70% at a concentration of 50 μM in activated polymorphonuclear leukocytes. This was confirmed by pyocyanin’s ability to hinder the production of compounds that require the enzymatic activity of 5-lipoxygenase for their production, such as 5-hydroxyeicosatetraenoic acid (5-HETE) and leukotriene B_4_ (Muller and Sorrell 1991). The 5-lipoxygenase inhibitory activity of pyocyanin assumes importance in neurodegenerative diseases since 5-lipoxygenase is upregulated in Alzheimer's disease and other age-associated neurological complications. Additionally, elevated levels of leukotriene B_4_ have also been associated with the exacerbation of neurodegenerative problems due to ageing, including Alzheimer’s disease (Chu and Praticò 2016). Thus, pyocyanin can be potentially used as a 5-lipoxygenase inhibitor in the treatment of neurodegenerative diseases such as Alzheimer’s disease. Fig. 9 schematically represents the mechanism underlying pyocyanin’s potential role as a neuroprotectant. However, a novel study has demonstrated the ability of pyocyanin to cross the blood-brain barrier and adversely affect cognitive function (Rashid et al. 2022). Thus, further studies must be conducted both in vitro and in vivo in order to elucidate the safety and viability of pyocyanin as a clinical neuroprotectant as well as its underlying mechanism of action.

### Pyocyanin as an antibacterial agent

**Fig. 10:**
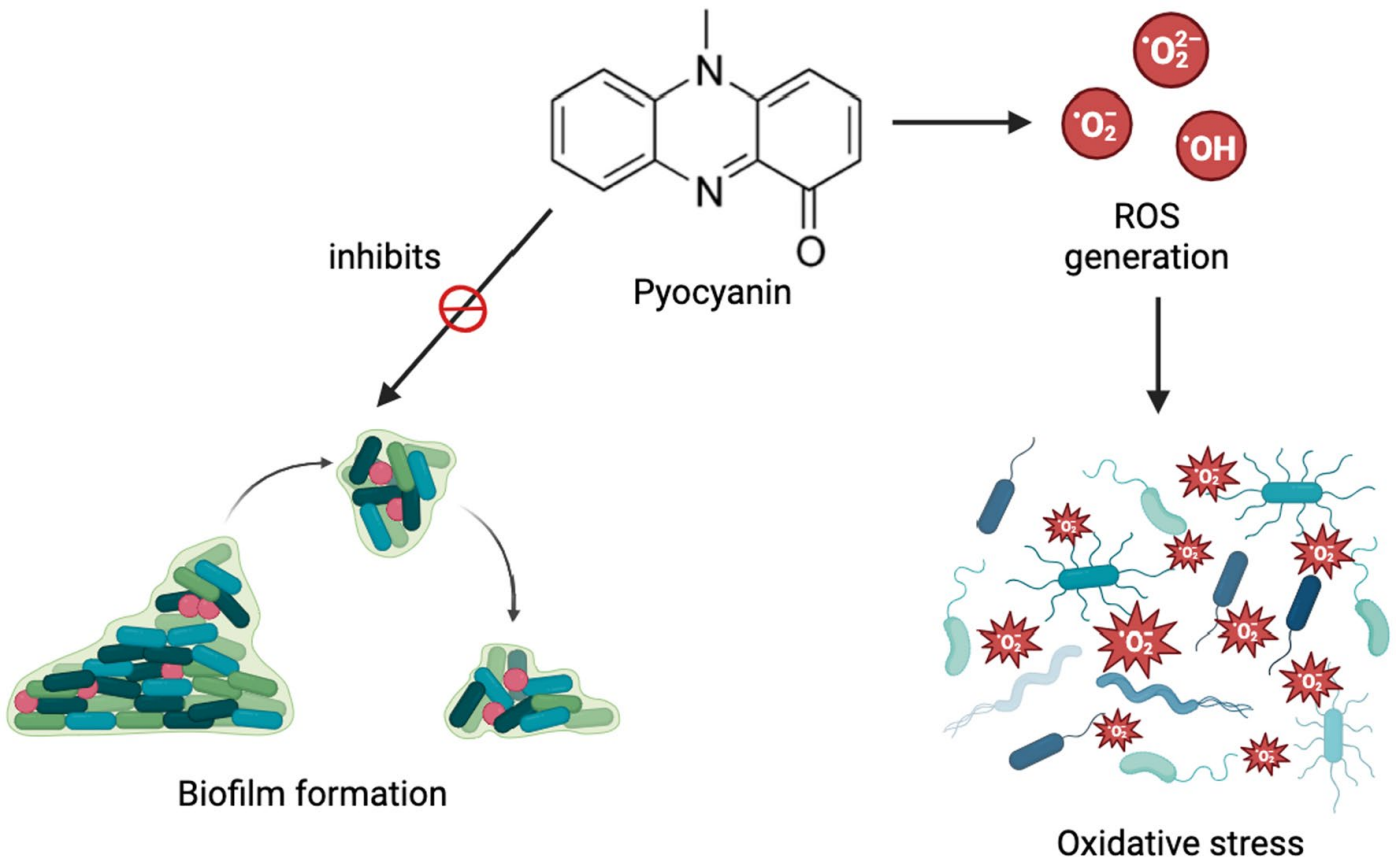
Antibacterial property of pyocyanin

Since several bacterial species have the ability to thrive in a range of conditions, bacterial infections are highly common in humans due to several types of exposure including contaminated food and water, direct contact, insect and animal bites, as well as airborne transmission through the inhalation of respiratory droplets and dust particles (Doron and Gorbach 2008). Pyocyanin is a potent antibacterial agent against both Gram-positive and Gram-negative bacteria, mainly because of its ability to create oxidative stress via ROS production. Moreover, pyocyanin inhibits bacterial pathogenesis by hindering biofilm formation which is necessary for bacterial survival and resistance. A study conducted by Laxmi et al. (2016) showed that pyocyanin was able to inhibit biofilm formation in six bacterial species, namely, *Bacillus* sp., *Bacillus altitudinis*, *Bacillus pumilus, Brevibacterium casei, Staphylococcus warneri,* and *Bacillus niacini* with an extremely low biofilm inhibitory concentration (BIC) of 9.51 pM. Though rare, *Brevibacterium casei* has been associated with peritonitis and brain abscesses while *Staphylococcus warneri* infections can lead to infective endocarditis, meningitis, UTIs, and vertebral discitis. *Bacillus* species have been implicated in several complications including osteomyelitis, endocarditis, bacteremia, burn infections, and meningitis. Though *Micrococcus luteus* and *Geobacillus stearothermophilus* were able to continue biofilm formation in the presence of pyocyanin, neither species is considered particularly harmful to humans except for possible nosocomial infections due to *Micrococcus luteus* (Laxmi et al. 2016). The antibacterial activity of pyocyanin has been depicted in Fig. 10.

**Fig. 11:**
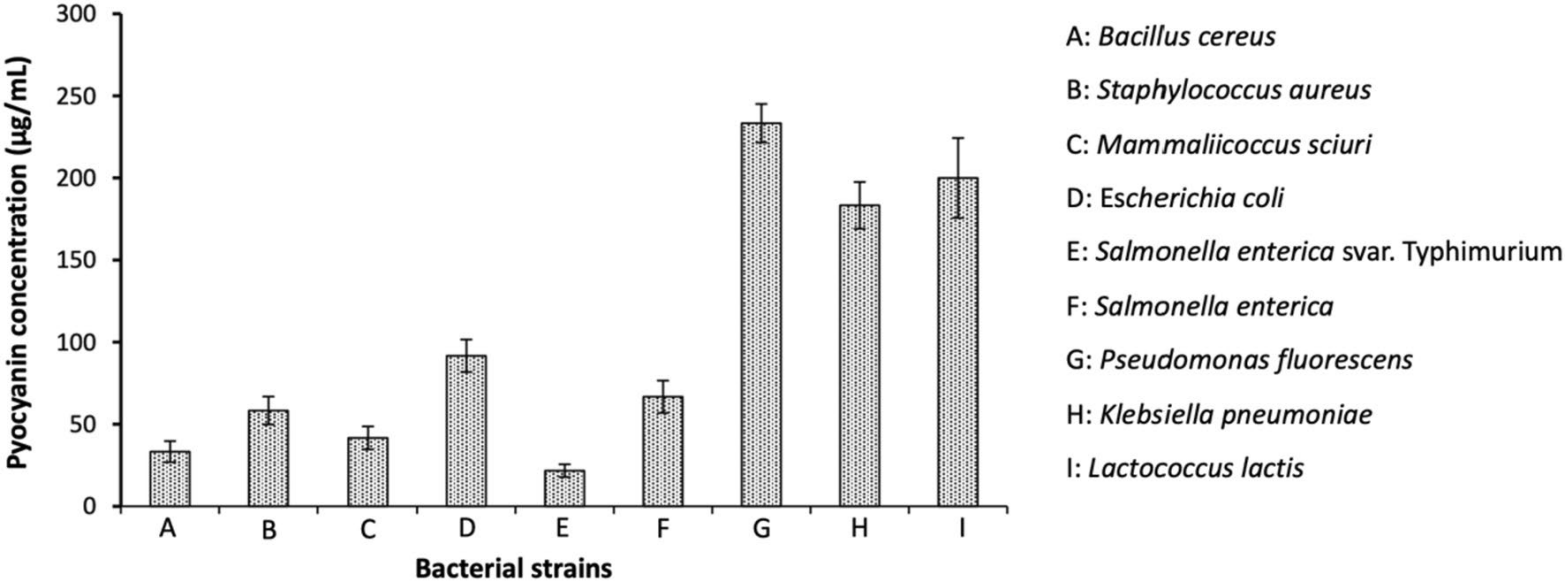
MIC of pyocyanin against different food-borne pathogenic bacteria (Hamad et al. 2020)

The concentration at which pyocyanin exhibits a higher antibacterial efficacy than tetracycline, a standard antibiotic, varies depending on the bacterial species, as reported by Hamad et al. (2020) based on the results of an experimental study. A concentration of 4.76 μM is sufficient for *Escherichia coli* while certain species including *Bacillus cereus*, *Salmonella enterica* svar. Typhimurium, and *Klebsiella pneumoniae* require a slightly higher concentration of 11.89 μM. While most strains of *E. coli* are harmless, certain strains do cause stomach infections leading to diarrhea and vomiting. More importantly, pyocyanin can be effective in the treatment of gastrointestinal infections caused by *B. cereus* and *S. typhimurium*, as well as bacterial pneumonia and urinary tract infections (UTIs) brought about by *K. pneumoniae*. In the case of *Staphylococcus aureus*, *Mammaliicoccus sciuri*, *Salmonella enterica*, and *Pseudomonas fluorescens*, a concentration of 23.78 μM is necessary for antibacterial activity, thus revealing the potential of pyocyanin in treating bacterial infections like salmonellosis, that are associated with these agents. However, at the same concentration, pyocyanin also inhibits *Lactococcus lactis* which is beneficial to the human immune system. It was observed that, of the aforementioned species, pyocyanin exhibits the highest inhibition against *Bacillus cereus*, closely followed by *Salmonella enterica* svar. Typhimurium (Hamad et al. 2020). Pyocyanin has also been shown to inhibit food-borne bacterial pathogens including Gram-positive *Bacillus spizizenii* and Gram-negative *Enterobacter aerogenes*, highlighting its potential as a food preservative (Saleem et al. 2021). The minimum inhibitory concentration (MIC) of pyocyanin against different food-borne pathogens has been graphically presented in Fig. 11. Gram-negative bacteria are reportedly less susceptible to pyocyanin as compared to Gram-positive bacteria (Devnath et al. 2017). It is important to note that *Pseudomonas aeruginosa* itself remains resistant to the antibacterial properties of pyocyanin (Özyürek et al. 2016). Experimental results published by Kamer et al. (2023) further demonstrated the ability of pyocyanin to exhibit antimicrobial activity against methicillin‐resistant *Staphylococcus aureus* (MRSA) strains that were highly unsusceptible to most antibiotics including oxacillin, cefoxitin, penicillin, gentamycin, and erythromycin. This is highly significant since MRSA is a major causative agent in hospital-acquired diseases and has been associated with prolonged infection and increased mortality (Lakhundi and Zhang 2018). Pyocyanin was able to inhibit all the aforementioned strains with an MIC of merely 38.05 μM. It was also able to significantly eradicate the biofilm formed by these MRSA strains in a concentration-dependent manner, destroying 29.7–56.8% of the biofilm at 47.56 μM with its anti-biofilm action increasing to 69–79.4% at 95.13 μM and finally, 83–88% at 190.26 μM (Kamer et al. 2023). While the currently available results are highly promising for the use of pyocyanin as an antibacterial, further research is necessary to ascertain the effect of pyocyanin on various beneficial bacteria such as *Lactobacillus* sp. and *Bifidobacterium* sp.

### Pyocyanin as an antifungal agent

**Fig. 12:**
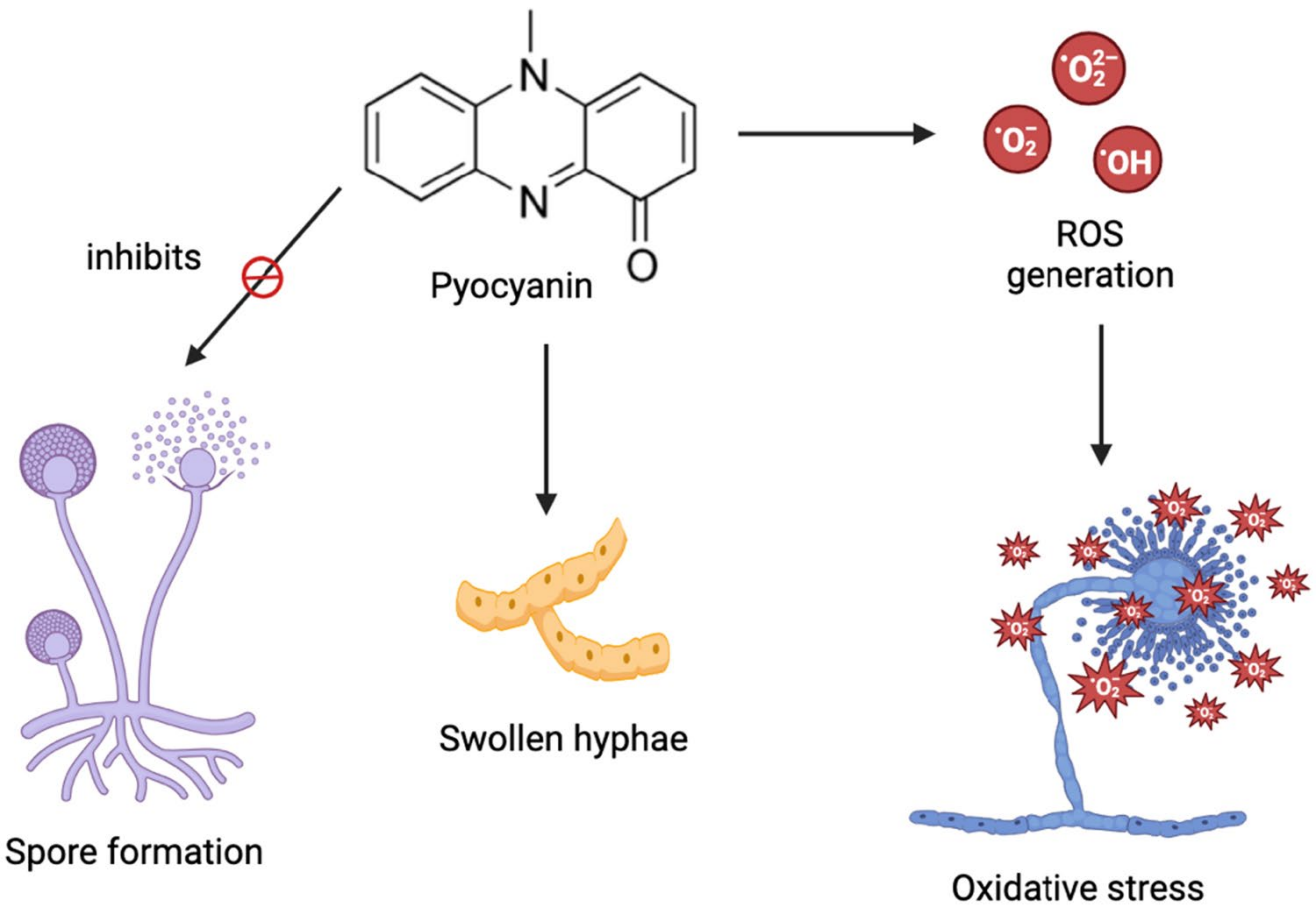
Antifungal activity of pyocyanin

Though fungal diseases are less prevalent in humans given the relatively small subset of fungal species that affect humans, changes in lifestyle along with the increasing human life span have put certain groups, especially immunocompromised individuals, at a high risk for certain harmful fungal infections (Gnat et al. 2021). Fungi that commonly infect humans and cause detrimental infections include *Candida* sp. and *Cryptococcus* sp. Pyocyanin has been shown to impede the growth of several fungal species to a significant extent. Pyocyanin acts against fungi in a concentration-dependent manner depending on the fungal species, showing increasing antagonism against *Aspergillus niger*, the causative agent of aspergillosis and aspergillomas, on increasing the concentration from 47.56 to 118.91 mM with no antifungal activity seen at 23.78 mM. In the case of *Candida albicans*, however, antifungal activity was detected starting from 23.78 mM. Not only is *C. albicans* responsible for oral and vulvovaginal candidiasis, but it can also lead to fatal systemic infections in immunocompromised individuals (Alzahrani and Alqahtani 2016). Pyocyanin is a potential treatment for fusariosis, onychomycosis, and keratitis as it demonstrates antifungal activity against *Fusarium oxysporum* at concentrations above 47.56 mM. Further, spore formation was seen to be inhibited and hyphae were swollen after 24 h of incubation as shown in Fig. 12 (Mahmoud et al. 2016).

As reported by Abdul-Hussien and Atia (2018), pyocyanin was able to inhibit the growth of *Aspergillus niger*, *Aspergillus fumigatus*, *Cryptococcus neoformans*, *Candida tropicalis,* and *Candida albicans* in the concentration range of 47.56 μM to 475.65 μM while *Candida krusei* was not as significantly affected. The anti-proliferative effect of pyocyanin against *C. neoformans* is especially important as it can potentially be translated into a treatment for cryptococcosis which leads to pneumonia and even meningitis, especially in immunocompromised patients (Abdul-Hussien and Atia 2018). Pyocyanin was also shown to be an effective treatment for tinea corporis, a fungal skin infection, due to its antifungal efficacy against *Trichophyton rubrum*, the fungal pathogen responsible for tinea corporis in humans. Pyocyanin had an MIC of 9.51 mM against *T. rubrum* which was lower than that of the commercially used standard, fluconazole, having an MIC value of 11.43 mM (El-Zawawy and Ali 2016). Another recent study published by Hamad et al. (2020) showed that pyocyanin possessed antifungal activity against *Aspergillus flavus, Aspergillus carbonarius, Aspergillus stynii, Fusarium verticillioides, Fusarium proleferatum, and Penicillium verrucosum* at a concentration of 475.65 μM, with the highest zone of inhibition observed against *Aspergillus stynii. A. flavus* is associated with skin infections including cutaneous aspergillosis, keratitis, and chronic granulomatous sinusitis which can potentially be treated using pyocyanin (Hedayati et al. 2007). *A. carbonarius*, on the other hand, is associated with the production of carcinogenic and immunosuppressive toxins like ochratoxin A (Llobregat et al. 2022). However, certain fungal species including *Aspergillus parasiticus*, *Aspergillus ochraceus*, *Aspergillus westerdijkiae,* and *Fusarium proliferatum* were able to grow in the presence of pyocyanin at the tested concentrations (Hamad et al. 2020). In order to further explore the use of isolated pyocyanin as an antifungal, the effect of pyocyanin on fungal pathogenesis in disease conditions as well as its adverse effects must be tested in vivo using animal models.

## Biomedical challenges due to pyocyanin

Though pyocyanin is associated with several medically beneficial properties, it is also actively involved in several biomedical challenges posed by *Pseudomonas aeruginosa* (Jabłońska et al. 2023). Pyocyanin’s ability to interfere with cellular functions in normal human cells exacerbates the effects of *P. aeruginosa* infections, especially in immunocompromised individuals, hospital patients, victims of burn wounds and other skin injuries, as well as those suffering from cystic fibrosis (CF).

### Pyocyanin in nosocomial infections

*Pseudomonas aeruginosa* is an opportunistic, pathogenic bacteria that is a prevalent cause of nosocomial infections in immunocompromised and hospitalized patients. These infections can be local or systemic in nature, ranging in severity, and have been linked to increased mortality rates (Ruffin and Brochiero 2019). Patients on ventilation are especially susceptible to infections affecting the respiratory system. Catalase is one of the important enzymes involved in cell signaling and maintaining optimum levels of hydrogen peroxide in the cell. O’Malley et al. (2003b) experimentally demonstrated the ability of pyocyanin to hinder the enzymatic activity of catalase in A549 human lung epithelial cells. This indicates the possibility that *P. aeruginosa* infections in immunocompromised patients can worsen lung function through increased oxidative stress. *P. aeruginosa* infections are also common in nosocomial urinary tract infections due to the use of urethral catheters, giving access to bacterial entry. As shown by an in vitro study conducted by McDermott et al. (2012), pyocyanin displayed cytotoxicity against RT4 human urothelial cells in a concentration-dependent manner from 25 to 50 μM leading to accelerated cell senescence which was also confirmed by a significant increase in the expression of senescence-associated-β-galactosidase. Thus, pyocyanin has been implicated in the deleterious effect of *P. aeruginosa* infections on normal urethral functioning in hospital patients. Further, it was shown that pyocyanin also increases prostaglandin E_2_ and IL-6 levels in RT4 cells thus suppressing inflammatory responses during urinary tract infections (McDermott et al. 2013). Though relatively less common, *P. aeruginosa* infection poses a risk to cardiac patients undergoing surgeries and other procedures as it can lead to bacterial endocarditis which is a lethal disease having a mortality rate of 20% (Polovina et al. 2014). Pyocyanin’s effect on the vascular system has been reported by Hempenstall et al. (2015) based on experimental studies conducted on porcine hearts. At physiological concentrations ranging from 0.1 to 10 μM, pyocyanin showed amplification of contractions in resting coronary arteries by inducing contractions of small amplitude, strengthening prostaglandin F_2α_-induced contractions, and inhibiting vascular relaxations even in the presence of relaxants such as neurokinin A, diethylamine nitric oxide, forskolin, dibuytyryl-cAMP, 8-bromo-cGMP, and P1075.

### Pyocyanin in cystic fibrosis

**Fig. 13:**
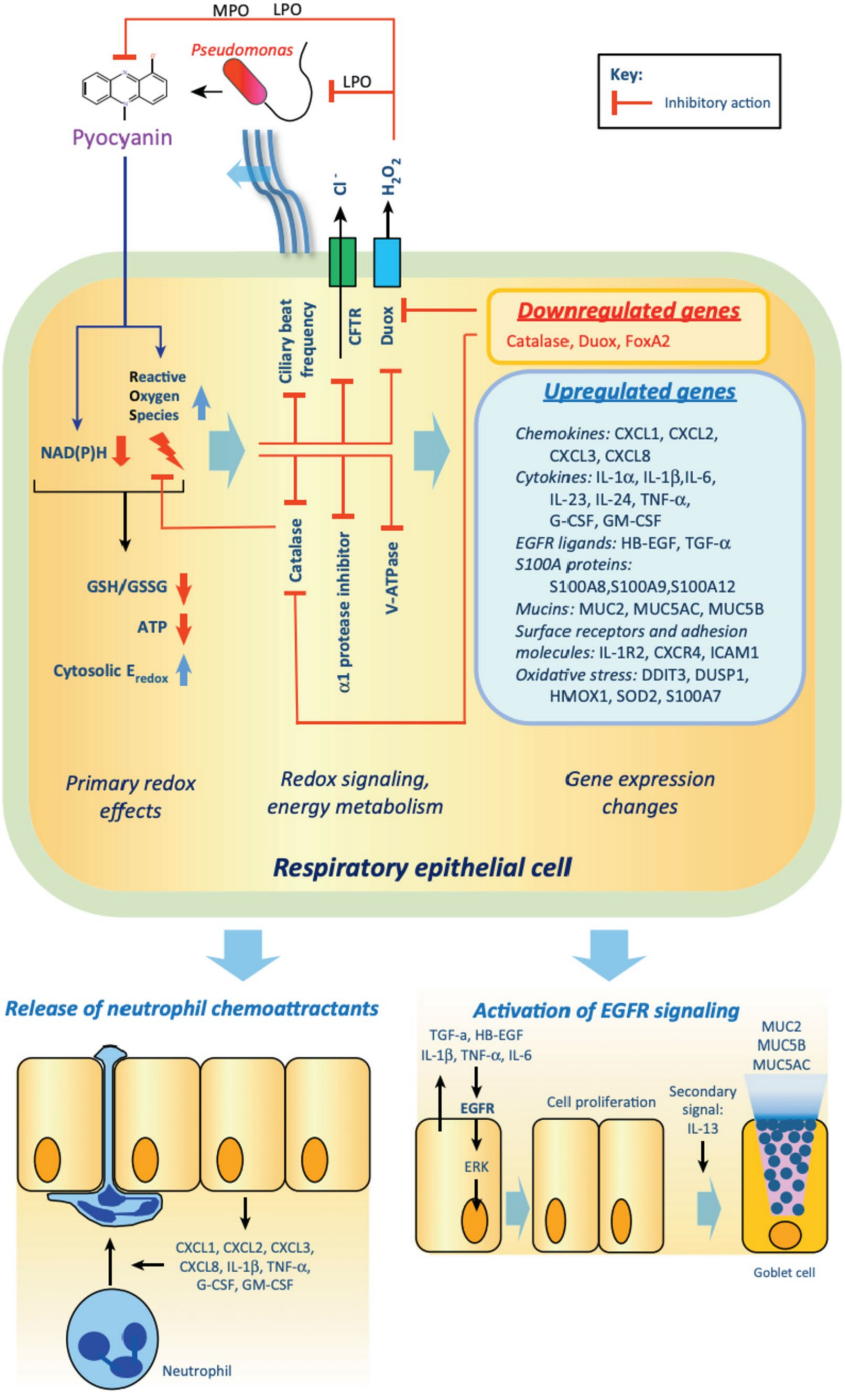
Mechanism of action of pyocyanin in the respiratory epithelium of CF patients (Rada and Leto 2013)

Cystic fibrosis (CF) is a life-threatening, genetic disorder in which mucus build-up in the lungs makes patients highly vulnerable to bacterial infections. *P. aeruginosa* is one of the most common causes of infection in the respiratory tract and the bacterial colonies produce large amounts of pyocyanin during CF pathogenesis (Lau et al. 2004b). In CF, there is a decrease in the expression of CF transmembrane conductance regulator (CFTR) anion transporters in tracheobronchiolar epithelial cells which are crucial for the host immune response. This leads to increased thickness of the mucus, lowering of the airway surface liquid volume, and increased osmolarity, due to which *Pseudomonas* bacteria can easily colonize and form biofilms to escape the host immune system’s attempts at clearing out the infection (Campodónico et al. 2008). Consequently, pyocyanin has been detected at significantly high levels of up to 100 μM in the lungs and sputum samples of patients suffering from CF (Bhargava et al. 2014). Kong et al. (2006) experimentally showed, both in vitro, using 16HBEo^–^ CFTR-expressing broncho-epithelial cells, and ex vivo, using primary nasal epithelial (PNE) cells, that pyocyanin can inhibit the activity of V-ATPase in a dose-dependent manner with significant inhibition recorded at a concentration of 128.42 μM. This, in turn, hinders the V-ATPase-dependent expression and localization of CFTR, an ion-channel protein required for proper lung function, thus leading to the exacerbation of CF. In the presence of flagellin, pyocyanin was shown to activate NF-κB, one of the primary regulators of inflammation, in CF airway epithelial cells. Pyocyanin-mediated activation of NF-κB is significant because chronic inflammation caused by NF-κB forms one of the main characteristics of CF lung infections (Schwarzer et al. 2008). Pyocyanin also disrupts calcium homeostasis through oxidative stress caused by the production of excess ROS via NADPH oxidation, as demonstrated by Winstanley and Fothergill (2009). Since the conformation of chloride ion channels is regulated by ATP levels, these channels are affected by the oxidative stress caused by pyocyanin which is over-produced by most *P. aeruginosa* strains found in CF infections. Pyocyanin further hinders cellular respiration and reduces intracellular levels of cAMP, NADPH, and ATP (Fothergill et al. 2007). Reduced ATP levels also inhibit ciliary motion, reduce cellular metabolism, and impair the functioning of CFTR channels. Reduction of NADPH levels not only impairs the antimicrobial activity of the respiratory cells by decreasing catalase expression but also hinders the ability of Duox1 and Duox2, epithelial NADPH oxidases, to inactivate pyocyanin. The effect of pyocyanin on the respiratory epithelium of CF patients has been diagrammatically elucidated in Fig. 13 (Rada and Leto 2013). Pyocyanin-producing strains are generally more persistent in the lungs of infected patients as compared to pyocyanin-deficient strains of *P. aeruginosa*, indicating that pyocyanin intensifies the severity of bacterial infections. Evidence-based results from a study conducted by Caldwell et al. (2009) demonstrated that clinical isolates of *P. aeruginosa* obtained from cystic fibrosis patients produced pyocyanin in greater amounts as compared to laboratory strains. Further, the results indicated that pyocyanin-exposed mouse lungs were more susceptible to several pulmonary complications that are common in CF patients including airway fibrosis, goblet cell hyperplasia and metaplasia, and destruction of the alveolar space.

### Pyocyanin in the impairment of wound healing

*Pseudomonas aeruginosa* has been reported to impair natural repair processes, thus impeding the efficiency of healing mechanisms, especially the restoration of epithelial integrity, after injuries, burns, and wounds (Ruffin and Brochiero 2019). *Pseudomonas aeruginosa* infections are common in epithelial skin barriers in contact with the external environment such as the respiratory tract and are especially prevalent in the cutaneous epithelium after damage due to chronic wounds such as burns or due to diabetes. Pyocyanin has been shown to induce senescence in A549 human lung epithelial cells due to oxidative stress as indicated by the increase in hydrogen peroxide production upon increasing the concentration of pyocyanin from 1 to 10 μM. This was further confirmed by the ability of glutathione, an antioxidant, to prevent the observed cell death in A549 cells (Muller 2006). An experimental study published by Muller et al. (2009) used an in vitro wound repair model to demonstrate pyocyanin’s concentration-dependent inhibition of wound repair. Pyocyanin was detected in infected wound dressings of burn patients and comparable concentrations of the compound were used to treat normal primary human diploid fibroblasts (HDFs). At concentrations ranging from 1 to 50 μM, pyocyanin was able to arrest the growth of treated HDFs (Muller et al. 2009). Further, the study showed that pyocyanin increased cell senescence in human skin fibroblasts of burn victims via oxidative stress which activates the Erk/p38 mitogen-activated protein kinase (MAPK) pathway (Alcorta et al. 1996). This upregulates the expression of p16 which is an inhibitor of pRb, a cell cycle regulator, thus resulting in the arrest of the cell cycle which ultimately causes premature senescence and prevents normal recovery (Muller et al. 2009). Another in vitro study conducted by Gonzalez et al. (2016) demonstrated the ability of *P. aeruginosa* to proliferate rapidly in burn wound exudates. An increased production of virulence factors, including pyocyanin, by *P. aeruginosa* was also detected as compared to the production rates generally observed in standard laboratory cultures. Increased virulence can contribute to burn wound sepsis which impairs recovery and may also lead to death in extreme cases.

### Pyocyanin in *Pseudomonas aeruginosa* infections

**Fig. 14:**
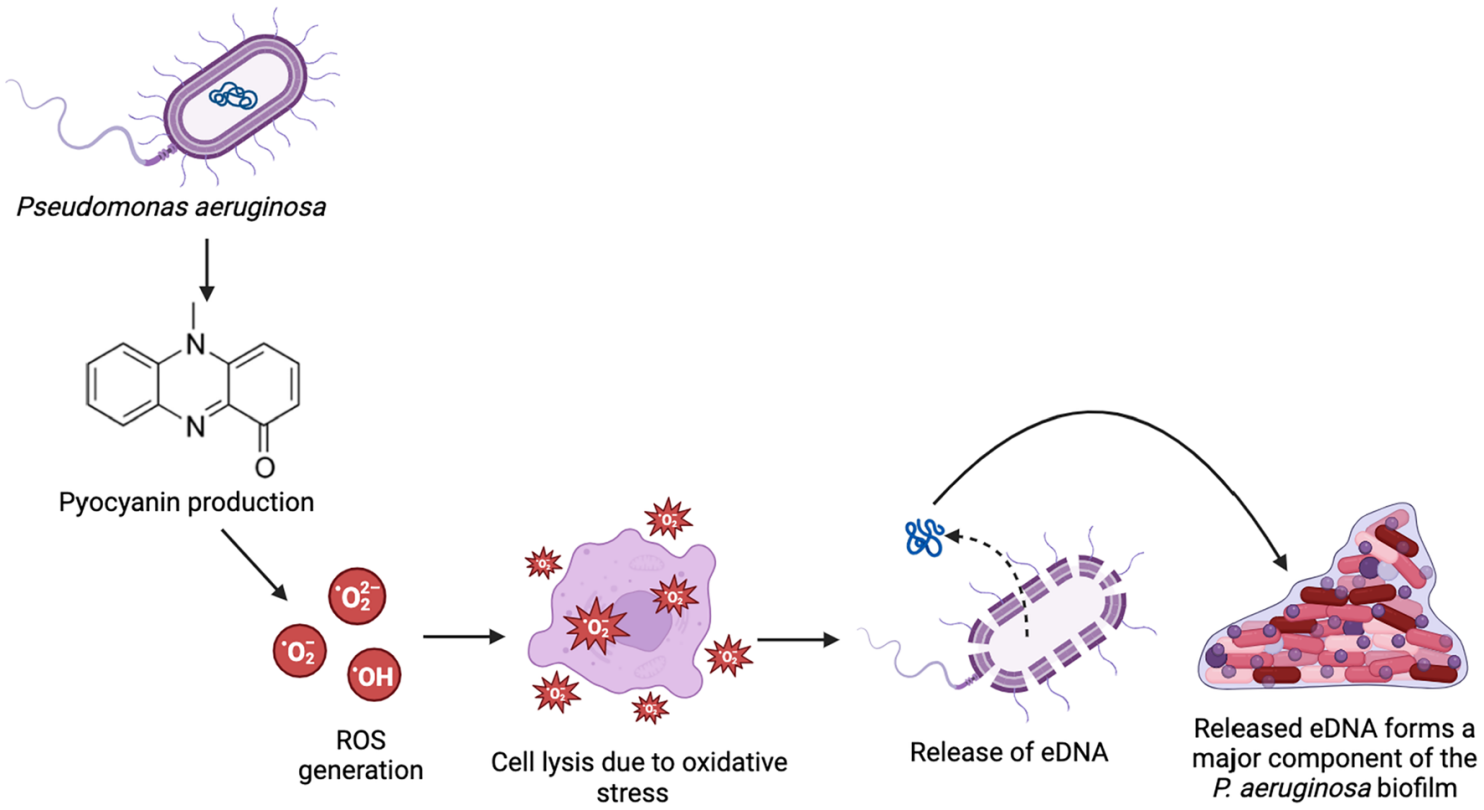
Pyocyanin-mediated release of eDNA via cell lysis due to oxidative stress

*Pseudomonas aeruginosa* is an opportunistic pathogen that has a complex pathophysiology and can affect a variety of hosts, including plants, humans, and insects (Lau et al. 2004a). Pyocyanin is one of the main virulence factors produced by *P. aeruginosa* (Bhargava et al. 2014). It has been implicated in several *P. aeruginosa* infections and has been reported to play a crucial role in exacerbating the severity and duration of these infections. Both in vitro and in vivo studies have shown the apoptotic effect of pyocyanin on neutrophils (Usher et al. 2002). Using adult CD-1 mice as animal models of lung infection, Lau et al. (2004b) revealed that the maximum level of virulence due to *P. aeruginosa* was recorded in the presence of pyocyanin, implying that pyocyanin is essential for the complete pathogenesis of *P. aeruginosa* infections. Moreover, the infected mice were unable to effectively clear out wild-type *P. aeruginosa* due to which its concentration was more than ten times that of the mutant, pyocyanin-deficient strains. These results were further supplemented by the experimental results published by Allen et al. (2005) showing that, in murine models, wild-type strains of *P. aeruginosa* that produce pyocyanin were unable to be cleared out as efficiently as phenazine-deleted strains that lacked the ability to produce pyocyanin. This was associated with the increased survival of the wild-type strains due to pyocyanin-induced suppression of the host’s neutrophilic inflammatory immune response to *P. aeruginosa* infections. In the case of wild-type bacterial infections, the neutrophil numbers as well as the concentrations of cytokines such as IL-6 and IL-1β, and chemokines like MIP-2 and KC, were seen to be significantly decreased. Pyocyanin also acts as a QS signaling molecule and has been shown to signal the upregulation of QS-associated genes (Dietrich et al. 2006). In this manner, pyocyanin facilitates biofilm formation which increases the survival and resistance of *P. aeruginosa* infections. eDNA is a major component of *P. aeruginosa* biofilms and its release via cell lysis is mediated by QS signaling molecules like pyocyanin, *Pseudomonas* quinolone, and *N*-acyl-l-homoserine lactone (AHL) as well as pili and flagella mechanisms (Das et al. 2016; Thees et al. 2021). Pyocyanin causes oxidative stress in the host cells via generation of ROS which cause damage to the DNA and important cell cycle components, deplete NADPH, disrupt the membrane potential, and inhibit essential enzymes. Thus, while pyocyanin is not essential for biofilm formation, it is associated with the accelerated lysis of infected host cells and the subsequent release of eDNA, as seen in Fig. 14 (Hall et al. 2016). Pyocyanin also contributes to biofilm formation by indirectly stimulating the release of extracellular polymeric substances (EPS) like polysaccharides, proteins, and lipids. Furthermore, it has been proposed that pyocyanin enhances biofilm expansion in oxygen-deficient conditions by accepting electrons to reduce oxidative stress (Meirelles and Newman 2018). However, phenazines like PCA and pyocyanin have also been seen to inhibit biofilm formation in oxidizing environments by influencing the protein RmcA to degrade bis-3′,5′-cyclic-dimeric-guanosine (di-GMP), a ubiquitous secondary messenger that regulates biofilm formation (Okegbe et al. 2017).

## Limitations of pyocyanin

Since the discovery of pyocyanin in 1860 followed by the first mention of its biomedical use as an antibiotic in 1942, a number of biomedically useful properties of pyocyanin have been uncovered including its anticancer, neuroprotective, antimicrobial, and antifungal activities (Fordos 1863; Waksman 1973). However, despite its vast potential to cure a number of diseases, pyocyanin is yet to be approved for clinical use in biomedical therapy due to several issues that are yet to be addressed. The main limitation hindering pyocyanin’s widespread use as a therapeutic molecule is its role as a virulence factor that enhances the pathogenesis of *P. aeruginosa* (Lau et al. 2004b; Allen et al. 2005). Through its activity as a QS sensing molecule, pyocyanin also upregulates QS-associated genes and enhances eDNA release via cell lysis, thereby aiding in biofilm formation which increases the severity and resistance of nosocomial infections caused by *P. aeruginosa* (Dietrich et al. 2006; Hall et al. 2016). Pyocyanin has been implicated in impaired urethral functioning and attenuated lung function due to *P. aeruginosa* infections in hospital patients (O’Malley et al. 2003b; McDermott et al. 2012, 2013). It has also been shown to impair wound healing which prevents its use in treating burns and other wounds infected with bacteria or fungi despite its antibacterial and antifungal properties (Alcorta et al. 1996; Muller et al. 2009). Moreover, pyocyanin’s antimicrobial activity is not limited to pathogenic microbes and can affect gut microbes and beneficial bacteria such as *Lactobacillus lactis* which aid in boosting immune responses (Hamad et al. 2020). Additionally, pyocyanin is majorly responsible for the debilitating effect of *P. aeruginosa* lung infections in CF patients. Not only does it enhance the persistence of *P. aeruginosa*, but it also disrupts the chloride ion channels via ROS production and leads to pulmonary complications like goblet cell hyperplasia (Caldwell et al. 2009; Winstanley and Fothergill 2009). Despite pyocyanin’s effective inhibitory activity against AChE and 5-lipoxygenase, its use as a neuroprotectant is limited by its ability to penetrate the blood–brain barrier which could potentially cause cognitive impairment due to reduced brain function (Rashid et al. 2022). Therefore, the various adverse effects of pyocyanin must be neutralized before its beneficial properties can be harnessed for use in biomedical therapy.

## Conclusion

In conclusion, pyocyanin has indeed earned its title as a ‘double-edged sword’. It exhibits several properties that can be channeled to bring about favorable outcomes in biomedical therapy. As an antibacterial, pyocyanin has the potential to treat complex bacterial diseases and inhibit several common bacterial infections. Its antimicrobial efficacy can be harnessed in the form of antibiotic treatments and alternative therapies. Pyocyanin also displays potent antifungal activity against several fungi and yeasts. It is a promising antifungal agent in the treatment of fungal infections such as aspergillosis, tinea corporis, and vulvovaginal candidiasis. Neurotherapy is another important area in which pyocyanin finds potential use. While further studies are required to confirm the neuroprotective potential of pyocyanin in the management of neurodegenerative symptoms, the preliminary results obtained so far are encouraging. Most significantly, pyocyanin has demonstrated cytotoxic action against several cancerous cell lines. These studies must be validated using animal models for different types of cancer before pyocyanin can be used in clinical trials for anti-cancerous therapies. However, despite the plethora of biomedical applications of pyocyanin, its deleterious effects cannot be overlooked. *Pseudomonas aeruginosa* infections are common in hospital patients due to their compromised immune system and increased bacterial exposure through surgical equipment, ventilators, catheters, and other pharmacopoeia. Pyocyanin negatively affects various systems including the respiratory system, urinary system, and vascular system in nosocomial infections. It also has the ability to cross the blood-brain barrier and can potentially affect the nervous system and hinder neurological functions leading to cognitive impairment. Further, pyocyanin is responsible for decreasing lung function and complicating pulmonary issues in cystic fibrosis patients. Another biomedical issue caused by pyocyanin is the impairment of wound healing by accelerating the senescence of epithelial cells, thus impeding the recovery of burn wounds and other external injuries. Finally, pyocyanin is a major virulence factor that amplifies the duration, resistance, and severity of *P. aeruginosa* infections, making it difficult for the host system to effectively clear out the bacterial load. Thus, while pyocyanin does have potential as a natural therapeutic compound, its detrimental effects pose a challenge to its clinical implementation as a biomedical strategy.

## Future perspectives

Effective measures must be developed to attenuate the damage caused by pyocyanin and inhibit its increased production during *P. aeruginosa* infections. By controlling the production of pyocyanin and strictly regulating its use, its advantageous properties can be harnessed while limiting its adverse effects. The dual nature of pyocyanin makes it an ideal molecule for further research. The exacerbation of several disease conditions caused by *P. aeruginosa* infections can be prevented by inhibiting pyocyanin production. To this end, novel anti-pyocyanin techniques must be developed and implemented for the treatment of conditions such as infected wounds, nosocomial infections, and cystic fibrosis. On the other hand, experimental studies are necessary to understand the exact mechanisms underlying the adverse effects of pyocyanin. This will aid in neutralizing its toxic effect while exploiting its beneficial properties for biomedical therapy. Pyocyanin in its natural form may not be safe for human use but with further research it could be possible to modify the pigment such that it specifically targets pathogenic or malignant cells without harming healthy human cells, thereby bringing about the desired therapeutic effect. Considering the plethora of diverse properties exhibited by pyocyanin, there is an exigent need to elucidate its mechanism action at the molecular and genetic levels in order to counter its debilitating effects and ultimately facilitate its implementation as a biotherapeutic agent.

## Data Availability

Data sharing is not applicable to this article as no datasets were generated or analyzed during the current study.
